# Altered resting-state dynamic functional brain networks in major depressive disorder: Findings from the REST-meta-MDD consortium

**DOI:** 10.1016/j.nicl.2020.102163

**Published:** 2020-01-07

**Authors:** Yicheng Long, Hengyi Cao, Chaogan Yan, Xiao Chen, Le Li, Francisco Xavier Castellanos, Tongjian Bai, Qijing Bo, Guanmao Chen, Ningxuan Chen, Wei Chen, Chang Cheng, Yuqi Cheng, Xilong Cui, Jia Duan, Yiru Fang, Qiyong Gong, Wenbin Guo, Zhenghua Hou, Lan Hu, Li Kuang, Feng Li, Kaiming Li, Tao Li, Yansong Liu, Qinghua Luo, Huaqing Meng, Daihui Peng, Haitang Qiu, Jiang Qiu, Yuedi Shen, Yushu Shi, Tianmei Si, Chuanyue Wang, Fei Wang, Kai Wang, Li Wang, Xiang Wang, Ying Wang, Xiaoping Wu, Xinran Wu, Chunming Xie, Guangrong Xie, Haiyan Xie, Peng Xie, Xiufeng Xu, Hong Yang, Jian Yang, Jiashu Yao, Shuqiao Yao, Yingying Yin, Yonggui Yuan, Aixia Zhang, Hong Zhang, Kerang Zhang, Lei Zhang, Zhijun Zhang, Rubai Zhou, Yiting Zhou, Junjuan Zhu, Chaojie Zou, Yufeng Zang, Jingping Zhao, Calais Kin-yuen Chan, Weidan Pu, Zhening Liu

**Affiliations:** a Department of Psychiatry, The Second Xiangya Hospital, Central South University, 139 Middle Renmin Road, Changsha, Hunan 410011, China; b Mental Health Institute, Central South University, Changsha, Hunan 410011, China; c Department of Psychiatry, Yale University School of Medicine, New Haven, CT 06511, USA; d Department of Psychology, Yale University, 2 Hillhouse Avenue, New Haven, CT 06511, USA; e CAS Key Laboratory of Behavioral Science, Institute of Psychology, Beijing 100101, China; f Department of Psychology, University of Chinese Academy of Sciences, Beijing 100049, China; g Magnetic Resonance Imaging Research Center and Research Center for Lifespan Development of Mind and Brain, Institute of Psychology, Chinese Academy of Sciences, Beijing 100101, China; h Department of Child and Adolescent Psychiatry, NYU School of Medicine, New York, NY 10016, USA; i Nathan Kline Institute for Psychiatric Research, Orangeburg, NY 10962, USA; j The first affiliated hospital of Anhui Medical University, Hefei, Anhui 230022, China; k Beijing Anding Hospital, Capital Medical University, Beijing 100088, China; l The First Affiliated Hospital of Jinan University, Guangzhou, Guangdong 510630, China; m Sir Run Run Shaw Hospital, Zhejiang University School of Medicine, Hangzhou, Zhejiang 310016, China; n Department of Psychiatry, First Affiliated Hospital of Kunming Medical University, Kunming, Yunnan 650032, China; o Department of Psychiatry, First Affiliated Hospital, China Medical University, Shenyang, Liaoning 110001, China; p Clinical Research Center and Division of Mood Disorders, Shanghai Mental Health Center, Shanghai Jiao Tong University School of Medicine, Shanghai 200030, China; q Huaxi MR Research Center, West China Hospital, Sichuan University, Chengdu, Sichuan 610041, China; r Department of Psychosomatics and Psychiatry, School of Medicine, Zhongda Hospital, Southeast University, Nanjing, Jiangsu 210096, China; s Department of Psychiatry, The First Affiliated Hospital of Chongqing Medical University, Chongqing 400016, China; t Mental Health Center, West China Hospital, Sichuan University, Chengdu, Sichuan 610041, China; u Department of Clinical Psychology, Suzhou Psychiatric Hospital, The Affiliated Guangji Hospital of Soochow University, Suzhou, Jiangsu 215137, China; v Faculty of Psychology, Southwest University, Chongqing 400716, China; w Department of Diagnostics, Affiliated Hospital, Medical School, Hangzhou Normal University, Hangzhou, Zhejiang 311121, China; x Department of Radiology, The First Affiliated Hospital, College of Medicine, Zhejiang University, Hangzhou, Zhejiang 310058, China; y National Clinical Research Center for Mental Disorders, Peking University Sixth Hospital, Beijing 100191, China; z Xi'an Central Hospital, Xi'an, Shaanxi 710003, China; Aa Department of Neurology, Affiliated Zhongda Hospital of Southeast University, Nanjing, Jiangsu 210009, China; Ab Department of Psychiatry, The Fourth Affiliated Hospital, College of Medicine, Zhejiang University, Hangzhou, Zhejiang 310058, China; Ac Institute of Neuroscience, Chongqing Medical University, Chongqing 400016, China; Ad Chongqing Key Laboratory of Neurobiology, Chongqing 400016, China; Ae Department of Neurology, The First Affiliated Hospital of Chongqing Medical University, Chongqing 400016, China; Af The First Affiliated Hospital of Xi'an Jiaotong University, Xi'an, Shaanxi 710061, China; Ag First Hospital of Shanxi Medical University, Taiyuan, Shanxi 030001, China; Ah Center for Cognition and Brain Disorders, Institutes of Psychological Sciences, Hangzhou Normal University, Hangzhou, Zhejiang 311121, China; Ai Zhejiang Key Laboratory for Research in Assessment of Cognitive Impairments, Hangzhou, Zhejiang 311121, China; Aj Department of Psychology, The University of Hong Kong, Hong Kong 999077, China; Ak Medical Psychological Institute, Second Xiangya Hospital, Central South University, Changsha, Hunan 410011, China

**Keywords:** Depression, Default-mode, FMRI, Dynamic functional connectivity, Connectome, Temporal variability

## Abstract

**Background:**

Major depressive disorder (MDD) is known to be characterized by altered brain functional connectivity (FC) patterns. However, whether and how the features of dynamic FC would change in patients with MDD are unclear. In this study, we aimed to characterize dynamic FC in MDD using a large multi-site sample and a novel dynamic network-based approach.

**Methods:**

Resting-state functional magnetic resonance imaging (fMRI) data were acquired from a total of 460 MDD patients and 473 healthy controls, as a part of the REST-meta-MDD consortium. Resting-state dynamic functional brain networks were constructed for each subject by a sliding-window approach. Multiple spatio-temporal features of dynamic brain networks, including temporal variability, temporal clustering and temporal efficiency, were then compared between patients and healthy subjects at both global and local levels.

**Results:**

The group of MDD patients showed significantly higher temporal variability, lower temporal correlation coefficient (indicating decreased temporal clustering) and shorter characteristic temporal path length (indicating increased temporal efficiency) compared with healthy controls (corrected *p* < 3.14×10^−3^). Corresponding local changes in MDD were mainly found in the default-mode, sensorimotor and subcortical areas. Measures of temporal variability and characteristic temporal path length were significantly correlated with depression severity in patients (corrected *p* < 0.05). Moreover, the observed between-group differences were robustly present in both first-episode, drug-naïve (FEDN) and non-FEDN patients.

**Conclusions:**

Our findings suggest that excessive temporal variations of brain FC, reflecting abnormal communications between large-scale bran networks over time, may underlie the neuropathology of MDD.

## 1 Introduction

Major depressive disorder (MDD) is a common psychiatric disorder characterized by deficits in regulating one's own emotions (Aldao et al., 2010; Anticevic et al., 2015). In MDD patients, one of the most notable changes revealed by functional magnetic resonance imaging (fMRI) is abnormalities in brain functional connectivity (FC) (Guo et al., 2014; Kaiser et al., 2015; Tao et al., 2013; Zhang et al., 2011a), which have been suggested as a potential mechanism underlying their emotional and cognitive symptoms (Marchetti et al., 2012; Whitfield-Gabrieli and Ford, 2012).

Traditional fMRI studies were performed under the assumption that pattern of brain FC remains stationary during the whole scanning period. Recently, however, it has been shown that brain FC fluctuates over time at sub-minute scales, which cannot be assessed by conventional static FC analysis methods (Chang and Glover, 2010; Hutchison et al., 2013). Therefore, “dynamic FC” has become a new topic in neuroimaging studies to track fluctuations in brain FC patterns (Preti et al., 2017). Notably, such fluctuations have been demonstrated to be involved in a wide range of cognitive and affective processes such as attention (Shine et al., 2016), learning (Bassett et al., 2011), executive functions (Braun et al., 2015), internally-oriented cognition (Zabelina and Andrews-Hanna, 2016) and mood (Betzel et al., 2017), as well as a number of common psychiatric disorders such as autism (Zhang et al., 2016), bipolar disorder (Nguyen et al., 2017) and schizophrenia (Dong et al., 2019; Guo et al., 2018). These findings highlight the importance of dynamic FC in understanding brain functions in both healthy and psychiatric populations.

Despite accumulating knowledge on dynamic features of brain FC, their relationships with MDD still remain unclear. Although a few studies (Demirtaş et al., 2016; Hou et al., 2018; Kaiser et al., 2016; Wei et al., 2017; Wise et al., 2017; Yao et al., 2019; Zheng et al., 2017) have started to investigate dynamic FC in MDD, these studies had several limitations. Firstly, the results reported from these studies are inconsistent. For example, while an earlier study (Demirtaş et al., 2016) found that MDD was related to decreased temporal variability of FC within the default-mode network (DMN), the opposite results were reported by two other studies (Kaiser et al., 2016; Wise et al., 2017). Such inconsistency may be partly due to relatively small sample sizes in these previous studies (see Supplemental Table S1 for a review), which could result in a relatively low reliability in neuroimaging studies (Button et al., 2013; Cao et al., 2019). Secondly, most previous studies were only focused on either fluctuations of FC within predefined regions of interests (ROIs) such as the medial prefrontal cortex (mPFC) (Kaiser et al., 2016; Wise et al., 2017), or changes in whole-brain connectivity states (Yao et al., 2019). However, characterizations of dynamic FC in MDD from both local and global perspectives remain poorly examined. Therefore, it is necessary to investigate global and local dynamics of FC in MDD with a larger well-powered sample to improve our understanding of this common mental disorder.

In this study, we aimed to characterize alterations of dynamic FC in MDD using a large, multi-site sample drawn from the REST-meta-MDD Project in China (Yan et al., 2019). To reach this goal, we used a novel dynamic network-based approach that allows us to identify altered dynamic FC patterns at both regional and global levels (Sizemore and Bassett, 2018). Specially, the brain is modeled as a multi-layer dynamic network, in which the layers represent brain FC patterns at different time points. Based on this model, metrics of several key spatio-temporal features of dynamic networks, including the temporal variability, temporal clustering and temporal efficiency, were estimated and compared between the MDD patients and healthy subjects. According to previous findings of the existence of altered dynamic FC in MDD (Kaiser et al., 2016; Wise et al., 2017), we hypothesize that MDD would disrupt the spatio-temporal organization of dynamic brain networks, leading to alterations in these metrics (e.g., increased temporal variability) in patients.

## 2 Methods and materials

### 2.1 Subjects

The analyzed sample consisted of 460 MDD patients and 473 healthy controls (HCs) recruited from 9 study sites across China, as a part of the REST-meta-MDD consortium (Yan et al., 2019). All subjects included were 18–65 years of age, with at least 5 years of education, and with an fMRI scan repetition time of 2 s and scan time ≥ 8 min. All patients met the Diagnostic and Statistical Manual of Mental Disorders-IV criteria for MDD (First et al., 1997), and had a total score ≥ 8 on the 17-item Hamilton Depression Rating Scale (HAMD) (Williams, 1988) at the time of scanning. Episodicity and medication information were available for a total of 372 patients from 6 sites, among which 155 were in their first episode of illness and had never taken antidepressants. Data on duration of illness were available for 382 patients from 7 sites. All study sites obtained approval from their local institutional review boards and ethics committees, and all participants provided written consent at their local institutions. See Tables 1 and 2 for sample details, and more details about the inclusion and exclusion criteria can be found in Supplemental Materials.

**Table 1:** The contributing sample size, clinical information of patients, and key data acquisition parameters of each site included in the current study. The nine sites were respectively located in: 1) the First Affiliated Hospital of Chongqing Medical University, Chongqing; 2) Affiliated Zhongda Hospital of the Southeast University, Nanjing; 3) the First Affiliated Hospital of Chongqing Medical University, Chongqing; 4) Anhui Medical University, Hefei; 5) Southwest University, Chongqing; 6) Beijing Anding Hospital of the Capital Medical University, Beijing; 7) the Second Xiangya Hospital of the Central South University, Changsha; 8) the West China Hospital of Sichuan University, Chengdu; 9) Affiliated Zhongda Hospital of the Southeast University, Nanjing.

Site	Samples	Clinical information of patients				MR Scanner	TR (ms)	TE (ms)	Time points
	MDD patients	HCs	HAMD score (mean ± SD)	Duration of illness/month (mean ± SD)^a^	FEDN/non-FEDN/unknown)				
1	19	5	24.632 ± 5.315	30.737 ± 56.854	13/6/0	GE 3T	2000	30	240
2	35	38	31.057 ± 5.401	36.571 ± 55.143	35/0/0	Siemens 3T	2000	25	240
3	41	41	20.659 ± 5.695	unavailable	0/0/41	GE 3T	2000	40	240
4	21	34	21.619 ± 7.110	99.095 ± 103.342	0/21/0	GE 3T	2000	22.5	240
5	221	226	21.448 ± 5.057	49.986 ± 64.932	96/117/8	Siemens 3T	2000	30	242
6	60	64	17.683 ± 6.485	90.344 ± 100.525	1/59/0	Siemens 3T	2000	30	240
7	21	20	23.762 ± 5.576	27.658 ± 25.833	0/0/21	Philips 3T	2000	30	250
8	24	29	21.583 ± 5.356	25.208 ± 29.123	10/14/0	Philips 3T	2000	30	240
9	18	16	10.111 ± 2.541	unavailable	0/0/18	Siemens 3T	2000	25	240

a Data on the duration of illness was available for only part of the patients. Abbreviations: MDD = major depressive disorder; HC= healthy control; FEDN = first-episode and drug-naïve; MR = magnetic resonance; TR = repetition time; TE = echo time.

**Table 2:** The demographic, clinical and image (head motion) characteristics of each group.

	Major depressive disorder (*n* = 460)	Health controls (*n* = 473)	Group comparisons
	(Mean ± SD)	(Mean ± SD)	
Age, years	36.785 ± 13.215	36.911 ± 15.252	*t* = −0.135, *p* = 0.892
Sex, male/female	155/305	177/296	*χ*^2^ = 1.412, *p* = 0.235
Education level, years	11.427 ± 3.235	12.980 ± 3.479	*t* = −7.061, *p* < 0.001
Mean FD	0.068 ± 0.033	0.070 ± 0.035	*t* = −0.866, *p* = 0.387
17-item HAMD scores	21.525 ± 6.642	/	/
Duration of illness, months^a^	54.311 ± 73.120	/	/

a Data on the duration of illness was available for 382 patients. Abbreviations: SD = standard deviation; FD = framewise-displacement; HAMD = Hamilton Depression Rating Scale.

### 2.2 Data acquisition, preprocessing and quality control

Resting-state fMRI and structural T1-weighted MRI brain scans were acquired at each site (see Table 1 for key data acquisition parameters) and were preprocessed using the DPARSF software (Yan, 2010) with a standardized protocol (Yan et al., 2019, 2016). To control for head motion and physiological noises, the Friston-24 head motion parameters, liner trends, as well as signals from the white matter, cerebrospinal fluid and whole brain were regressed out from the images (Friston et al., 1996; Laumann et al., 2017; Lydon-Staley et al., 2019). Subjects with poor image quality or excessive head motion (mean framewise-displacement (FD) (Jenkinson et al., 2002) > 0.2 mm) were excluded from analysis. See Supplemental Materials for further details.

### 2.3 Construction of dynamic brain network

**Fig. 1:**
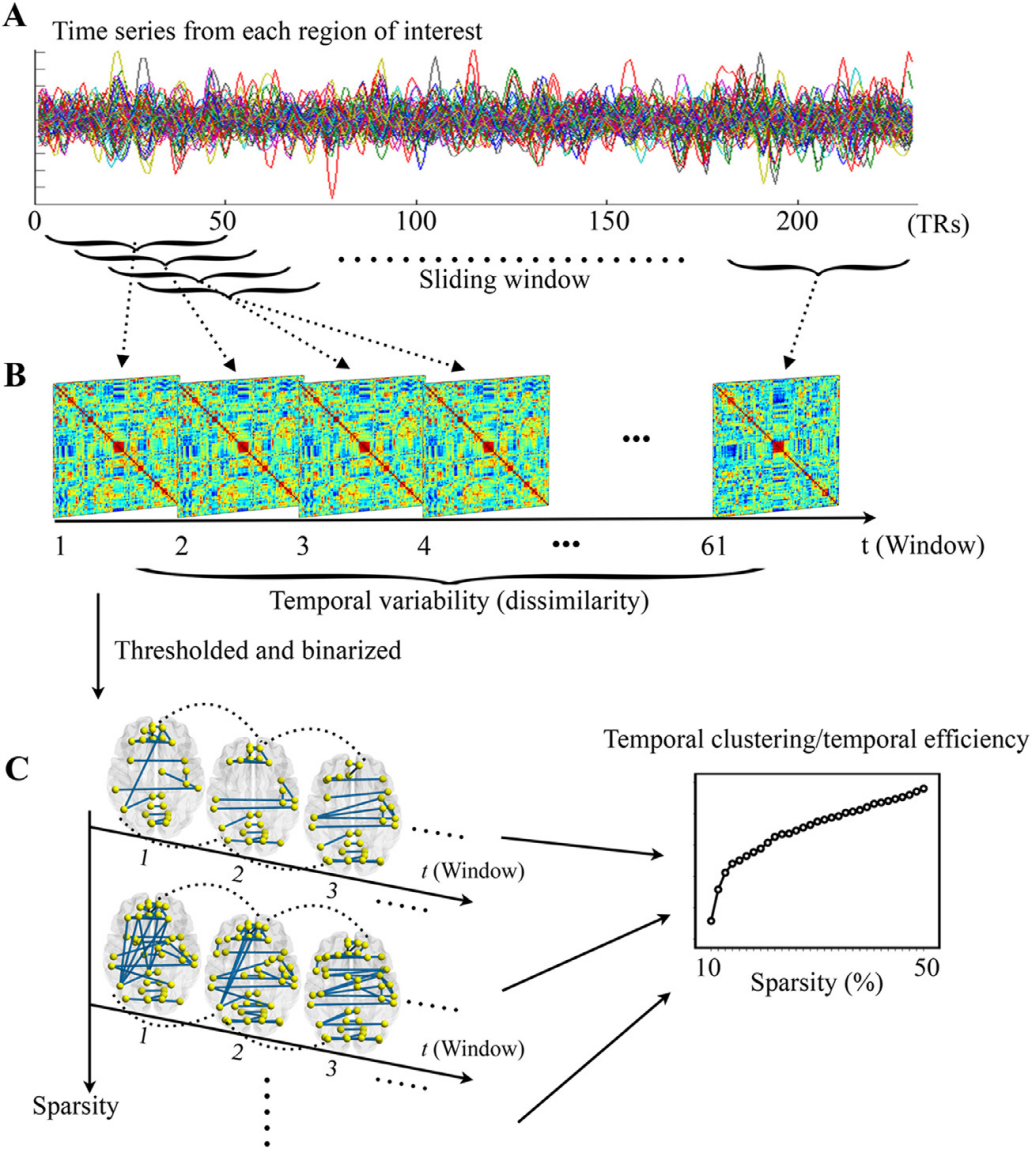
The procedures for constructing dynamic brain networks and computing dynamic network metrics. (**A**) The time series for all nodes were divided into a number of continuous time windows. (**B**) The whole-brain connectivity matrices were calculated within each window to compose a dynamic network, whose temporal variability was then estimated by average dissimilarities between different windows. (**C**) The dynamic brain networks were further thresholded and binarized with a range of sparsities from 10% to 50%, at which temporal clustering and temporal efficiency were estimated. TR = repetition time.

As summarized in Fig. 1, multi-layer dynamic brain networks were constructed using a widely-used sliding-window approach (Laumann et al., 2017; Reinen et al., 2018; Sun et al., 2019b) with nodes defined by the 160 ROIs in the Dosenbach functional atlas, which were derived from previous meta-analyses (Dosenbach et al., 2010) (see Supplemental Table S2 for list of ROIs). The mean time series of each of the 160 nodes were firstly extracted and divided into a number of continuous time windows (Fig. 1**A**). According to the previous recommendations (Leonardi and Van De Ville, 2015; Sun et al., 2019b; Zalesky and Breakspear, 2015), a window length of 100 s and a step length of 6 s were used in the primary analyses. This produced a total of 61 windows, and the whole-brain connectivity matrices were then calculated within each window using pairwise Pearson correlations. As a result, a multi-layer dynamic network *G* = (*G_t_*)_*t* =1,2,3,…,61_, where *G_t_* is the layer representing brain FC within the *t*th time window, was obtained for each subject (Fig. 1**B**). See Supplemental Materials for more details.

### 2.4 Dynamic brain network metrics

After constructing dynamic networks, we estimated several key spatio-temporal features of dynamic brain networks, including the temporal variability, temporal clustering and temporal efficiency, described as following:

*Temporal variability*: The variability of a dynamic brain network over time was measured by the average dissimilarity of its network structures between different time windows (Dong et al., 2019; Hou et al., 2018; Zhang et al., 2016). This was computed at both the global (*temporal variability*) and regional (*nodal temporal variability*) levels. Both of the measures range from 0 to 2, and a higher value indicates a higher variability.

*Temporal clustering:* Temporal clustering measures the consistency of each node's connected neighbors between consecutive time points, named the *temporal correlation coefficient* and *nodal temporal correlation coefficient* at global and regional levels, respectively (Sizemore and Bassett, 2018; Tang et al., 2010). They both range from 0 to 1, and a higher value indicates a higher consistency (higher temporal clustering).

*Temporal efficiency:* Temporal efficiency quantifies how quickly information can transmit between nodes in a dynamic network, named the *characteristic temporal path length* for the whole-brain and *nodal temporal path length* for each node (Sizemore and Bassett, 2018). These two measures range from 1 to infinite, with a lower value representing a shorter average temporal distance between nodes and a shorter time for information to be transferred between nodes on average (higher temporal efficiency) (Sizemore and Bassett, 2018; Thompson et al., 2017).

Since temporal clustering and temporal efficiency are only defined for binary networks (Sizemore and Bassett, 2018), we obtained binary dynamic networks by preserving only a particular proportion (“sparsity”) of the strongest connections between nodes on the FC matrices of each window (Fig. 1**C**). The metrics of temporal clustering and temporal efficiency were computed across a wide range of sparsities from 10% to 50% with an increment interval of 1%, to ensure that results were biased by a single sparsity level (Sreenivasan et al., 2017; Zhang et al., 2019; Zhang et al., 2011b). More details about these metrics and methods can be found in Supplemental Materials and Supplemental Fig. S1-S2, as well as a previous publication (Sizemore and Bassett, 2018).

### 2.5 Statistics

Temporal variability of the dynamic brain network was compared between the MDD and HCs groups by analysis of covariance (ANCOVA), where group was included as dependent variable, covarying for age, sex, education, mean FD and site. Similarly, the temporal correlation coefficient and characteristic temporal path length were compared using repeated-measures ANCOVA models, in which sparsity level (10% to 50%) was included as within-subject factor and group as a between-subject factor, with the same above covariates. We further investigated their associations with HAMD scores and duration of illness (when available) in patients using partial Spearman rank correlations, adjusting for age, sex and site. The temporal correlation coefficient and characteristic temporal path length were averaged across all sparsities before the correlation analyses. Significance was set at *p* < 0.05 after Benjamini-Hochberg false discovery rate (FDR) corrections across the three measures.

When significant between-group differences were detected on any of the examined dynamic network metrics, we further investigated which regional changes might particularly drive those effects. For that we compared the corresponding nodal metrics between groups for each of the 160 ROIs, using the same above ANCOVA or repeated-measures ANCOVA models. Similarly, their associations with the HAMD scores and duration of illness were examined using the partial Spearman rank correlations adjusting for age, sex and site. Significance was set at *p* < 0.05 after FDR corrections across the 160 ROIs. Results were visualized using the BrainNet Viewer (Xia et al., 2013).

### 2.6 Subgroup analyses

Subgroup analyses were performed to explore the possible influences of illness episodes and medication. Here, the MDD patients were divided into subgroups of first-episode, drug-naïve (FEDN) (*N* = 155) and non-FEDN (*N* = 217) patients, whose demographic and clinical information can be found in Supplemental Table S3. The metrics showing significant between-group differences were further compared between each pair of subgroups (FEDN vs non-FEDN, FEDN vs HCs, and non-FEDN vs HCs) using the same above ANCOVA or repeated-measures ANCOVA models; partial correlations were performed in each subgroup of patients separately, too. All analyses were FDR-corrected for number of tests (e.g., 3 metrics multiplied by 3 subgroup comparisons).

### 2.7 Validation analyses

To validate our findings, we additionally performed several auxiliary analyses as follows (see details in Supplemental Materials):

*Impact of parcellation schemes:* The entire analysis was rerun with a different parcellation scheme based on the automated anatomical labeling (AAL) atlas (Tzourio-Mazoyer et al., 2002) with 90 ROIs.

*Sliding-window lengths:* A set of different window and step lengths (window/step = [40, 60, 80, 100]/[4, 6, 8] seconds) were utilized in constructing dynamic networks to estimate the reproducibility of results across different analysis parameters.

*Subset analyses:* To evaluate whether the results were affected by sample population or unmatched education levels, group differences on each metric were tested within each of the following subsets: 1) the subsets of each individual site; 2) two split-half subsets randomly split from the whole sample; and 3) a subset extracted from the whole sample where education levels were matched between groups, by excluding all healthy subjects with years of education ≥ 16 or age ≥ 60. See Supplemental Table S4 for demographic and clinical information of each subset.

### 2.8 Data and code availability statement

Data of the REST-meta-MDD project are available at: http://rfmri.org/REST-meta-MDD. The dynamic network metrics were computed by a publicly-available MATLAB toolbox (https://github.com/asizemore/Dynamic-Graph-Metrics).

## 3 Results

### 3.1 Group comparisons and correlations

**Fig. 2:**
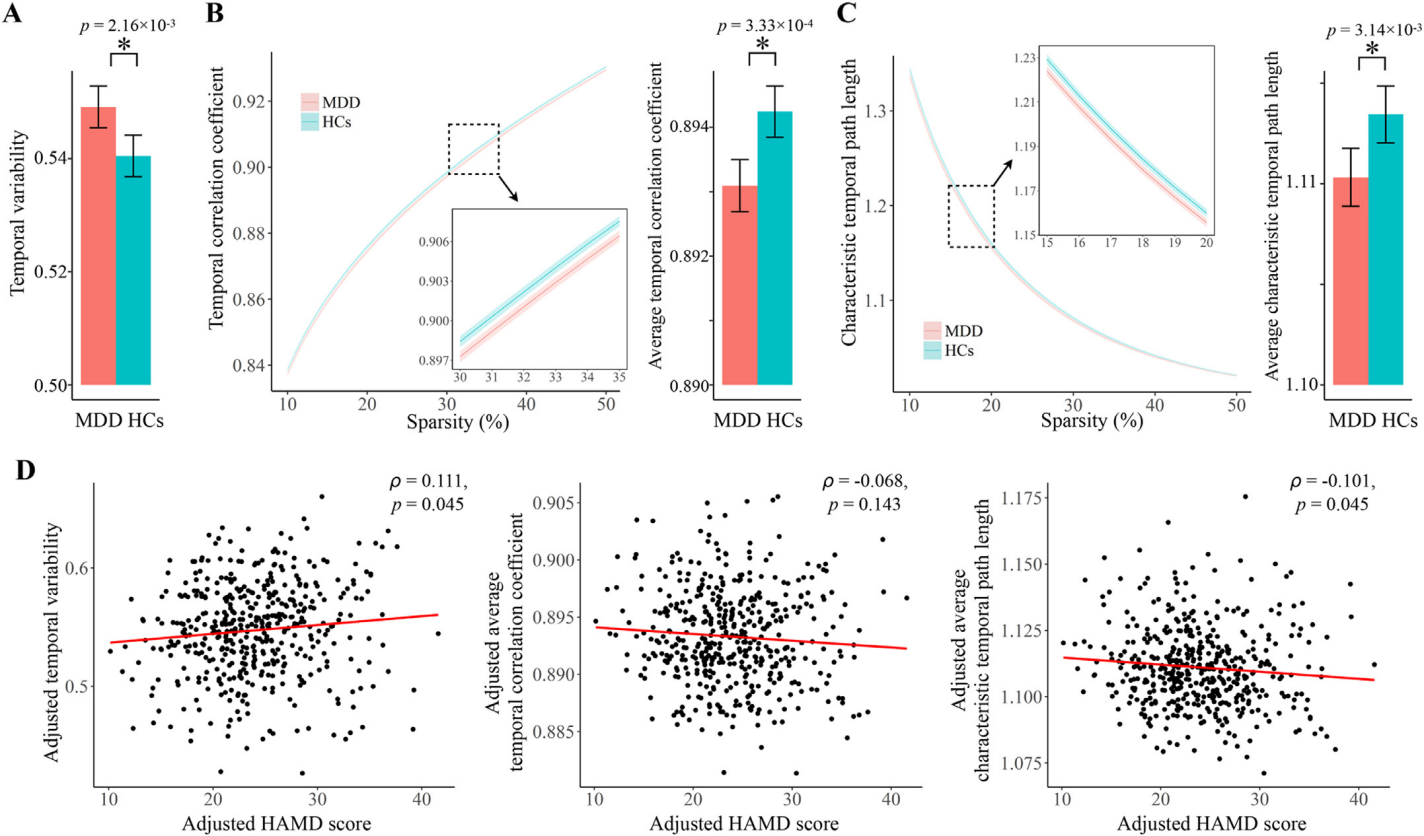
(A) Group comparison on temporal variability. (B-C) Group comparisons on the temporal correlation coefficient and characteristic temporal path length, with values at each sparsity level and average values across all sparsities (10% to 50%) both presented. (**D**) Partial correlations between each metric and the HAMD score, adjusted for age, sex and site effects. The error bars and shadows in (**A**)–(**C**) represent 95% confidence intervals, and all reported *p* values were corrected for multiple tests using the FDR method.

As shown in Fig. 2**A**-**C**, the MDD group showed a significantly higher temporal variability (*F* = 10.218, FDR-corrected *p* = 2.16×10^−3^), a significantly lower temporal correlation coefficient (*F* = 15.071, FDR-corrected *p* = 3.33×10^−4^), and a significantly shorter characteristic temporal path length (*F* = 8.768, FDR-corrected *p* = 3.14×10^−3^) compared with HCs. The differences in temporal correlation coefficient and characteristic temporal path length were significant at all sparsity levels (*p* < 0.05, Supplemental Table S5). Moreover, the temporal variability and characteristic temporal path length were found to be significantly correlated with the HAMD scores in patients (Spearman's rho = 0.111 and −0.101, FDR-corrected *p* = 0.045 and 0.045 for temporal variability and characteristic temporal path length, respectively, Fig. 2**D**), while no significant correlations were found between any metrics and duration of illness (FDR-corrected *p* > 0.05).

**Fig. 3:**
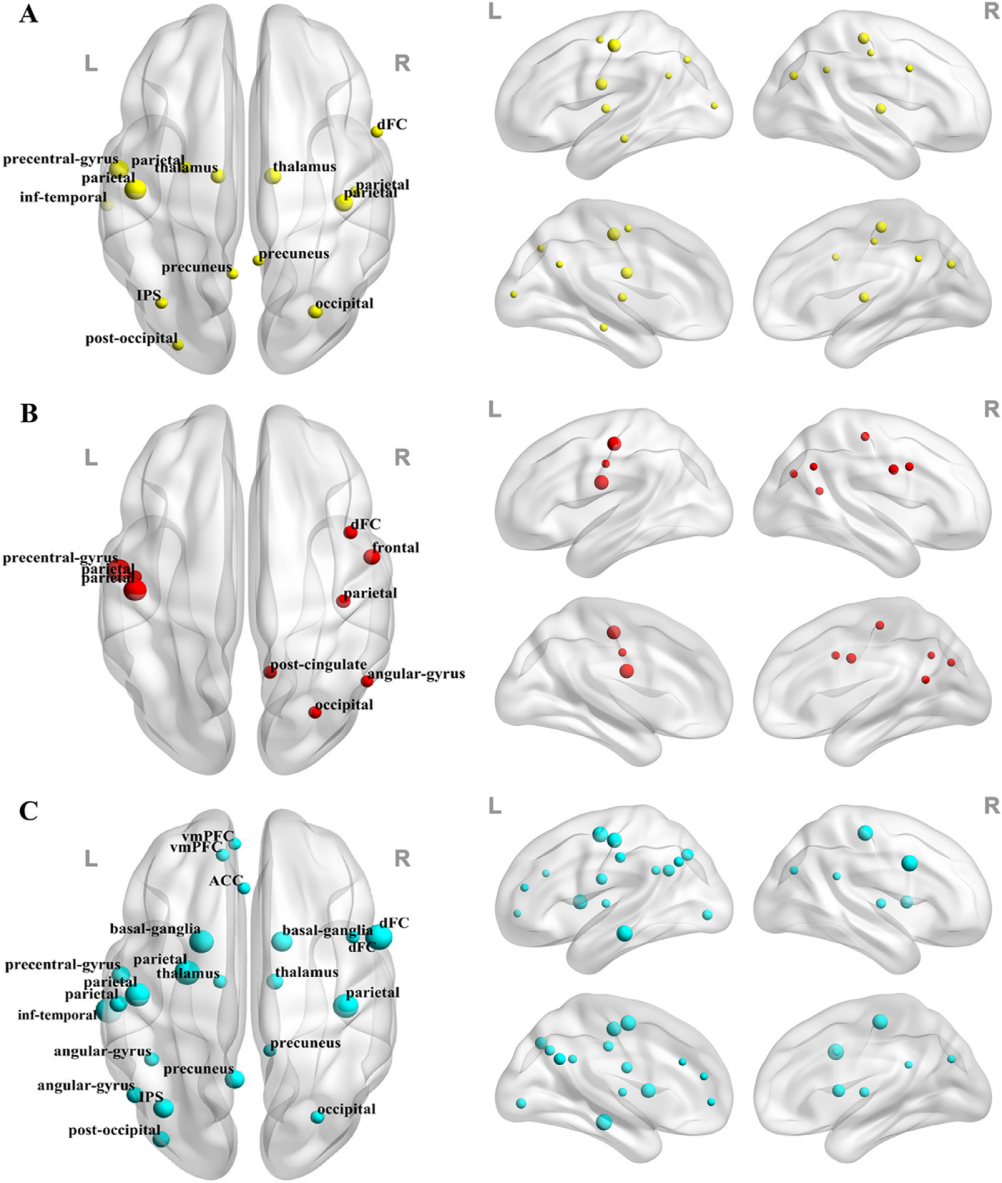
The nodes showing (**A**) a higher nodal temporal variability, (**B**) a lower nodal temporal correlation coefficient, and (**C**) a shorter nodal temporal path length in MDD patients than HCs (with FDR-corrected *p* < 0.05). Sizes of plots are weighted by *F* values. ACC = anterior cingulate cortex; dFC = dorsal frontal cortex; IPS = intraparietal sulcus; *L* = left hemisphere; *R* = right hemisphere; vmPFC = ventromedial prefrontal cortex.

At regional level, significantly increased nodal temporal variability, decreased nodal temporal correlation coefficient and decreased nodal temporal path length were found in a total of 14, 9, and 22 ROIs, respectively (FDR-corrected *p* < 0.05). These ROIs highly overlapped among the three metrics and were chiefly located in the DMN (mPFC, precuneus, anterior/posterior cingulate gyrus, angular gyrus, and inferior temporal cortex), the sensori-motor cortex (frontal and parietal areas), and the subcortex (thalamus and basal ganglia) (Fig. 3 and Supplemental Table S6). No correlations at the ROI level survived FDR correction (corrected *p* > 0.05).

### 3.2 Subgroup analyses

**Fig. 4:**
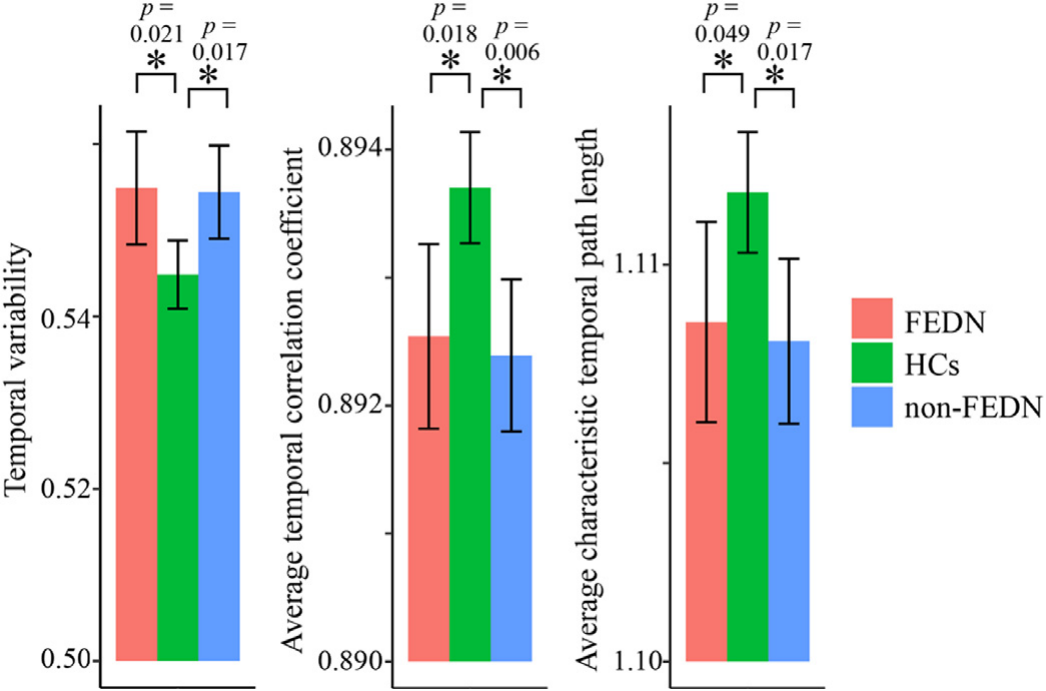
Results of subgroup comparisons among the first-episode, drug-naïve (FEDN) patients, non-FEDN patients and healthy controls (HCs). The error bars represent 95% confidence intervals, and all reported *p* values were corrected for multiple tests using the FDR method.

As shown in Fig. 4, both the subgroups of FEDN and non-FEDN patients showed significantly higher temporal variability, lower temporal correlation coefficient and shorter characteristic temporal path length compared with HCs (FDR-corrected *p* < 0.05), while no significant differences were found for any metrics between the FEDN and non-FEDN patients (FDR-corrected *p* > 0.05). Although no correlations in any subgroup survived FDR correction (corrected *p* > 0.05), trend-level effects with the same directionalities as observed in the overall patient sample were still present in both the FEDN and non-FEDN patients, suggesting that these correlations are not driven by illness duration or medication (Supplemental Fig. S3). However, the effect sizes in subgroups were relatively small and thus larger samples may be required in order to detect such effects.

### 3.3 Validation analyses

Using the AAL atlas, similar results were obtained at both global and regional levels (Supplemental Fig. S4). Similar results were also consistently found when repeating the analyses using different window/step lengths in constructing dynamic networks (Supplemental Table S7).

Significantly (or trend-level) higher temporal variability, lower temporal correlation coefficient and shorter characteristic temporal path length in MDD patients than HCs were consistently observed in 7 of the 9 sites (Supplemental Fig. S5). Such results were also consistently observed in two split-half subsets, and in the education-matched subset extracted from the whole sample (Supplemental Table S8).

## 4 Discussion

In the present study, using one of the largest MDD fMRI samples to date, we investigated resting-state dynamic FC in MDD using a novel dynamic network-based approach. The results revealed that MDD was associated with altered spatio-temporal organizations of dynamic brain networks, including increased temporal variability, decreased temporal clustering and increased temporal efficiency at both global and local levels. These results highlight the potential importance of dynamic brain network reconfiguration in neural mechanisms underlying MDD.

We found that MDD patients had a significantly higher temporal variability in organizations of their resting-sate functional brain networks compared with healthy subjects (Fig. 2**A**). This finding is in line with most previous studies (Hou et al., 2018; Kaiser et al., 2016; Wise et al., 2017) suggesting excessive fluctuations of brain FC in MDD patients during rest, yet in conflict with one study (Demirtaş et al., 2016). Recent work has shown that pooling data across different sites is an effective way to boost statistical power at only minimal cost of reliability loss in large-scale neuroimaging consortiums, especially when the total sample size > 250 (Cao et al., 2019). Therefore, our findings from the large multi-site sample with > 900 subjects may provide greater power than any of the previous studies mentioned above (Hou et al., 2018; Kaiser et al., 2016; Wise et al., 2017), offering more solid evidence that the brain FC patterns in MDD patients are temporally more variable during rest than those in healthy subjects.

Besides increased temporal variability, the MDD group showed a significantly decreased temporal correlation coefficient, which indicates lower consistency of FC patterns between consecutive time points (decreased temporal clustering) (Sizemore and Bassett, 2018). Normal brain FC patterns have been found to maintain relatively stability over periods of time, which is known as “FC states” possibly reflecting different cognitive or emotional states (Allen et al., 2014; Preti et al., 2017; Zalesky et al., 2014). The decreased temporal clustering may indicate a disruption of such features in the brain due to excessive fluctuations of FC. The MDD patients also showed increased temporal efficiency as characterized by a significantly decreased characteristic temporal path length, which suggests shortened delays for information transfer between nodes (Sizemore and Bassett, 2018; Thompson et al., 2017). While speculative, such alteration may be attributed to increased aberrant brain connections, which play roles as “shortcuts” beyond the common periodic transitions across relative stable FC states (Betzel et al., 2016; Zalesky et al., 2014), and thus may be another reflection of increased fluctuations in brain network structures.

The aberrant connections beyond normal successive FC states may interfere with meaningful interconnectedness for cognitive and mental processing (Betzel et al., 2016; Sun et al., 2019b; Zalesky et al., 2014). Moreover, the excessive reconfigurations of FC patterns may carry extra metabolic costs (Shine et al., 2018). Hence, all observed alterations in dynamic brain networks may together suggest less optimal information processing and a shift of economically balanced metabolic cost in the brains of MDD patients. Notably, these alterations were found in both FEDN and non-FEDN patients, and all of them showed significant (or trend-level) correlations with HAMD scores in the overall patient sample (Fig. 2**D**), suggesting that they are likely to be associated with the severity of depression symptoms rather than an epiphenomenon of illness duration and pharmacological effects.

In MDD patients, brain regions showing significant alterations at the nodal level were mostly distributed in the DMN, including the mPFC, anterior/posterior cingulate cortex, angular gyrus and precuneus (Fig. 3). These results are consistent with some previous studies, which have reported that MDD is related to excessive fluctuations of FC in DMN-related regions (Kaiser et al., 2016; Wise et al., 2017). It has been postulated that increased temporal variability of FC within the DMN may be associated with higher frequencies of spontaneous, internally-oriented cognition such as mind-wandering and creative thinking (Christoff et al., 2016; Kucyi and Davis, 2014; Sun et al., 2019a; Zabelina and Andrews-Hanna, 2016). In line with this interpretation, excessive fluctuations of FC in the DMN regions may reflect a failure to effectively control exaggerated internally-focused thoughts. These alterations, therefore, may be related to rumination, which is one of the core features of MDD defined as repetitive and passive focus on one's distress (Nolen-Hoeksema et al., 2008), although we are uncertain if they are the cause or the consequence of depression-related rumination. Prolonged self-referential emotional processing may further interfere with normal cognitive functions in MDD patients (Korgaonkar et al., 2014). Our hypotheses are supported by recent findings that temporal variabilities of FC within the DMN (Wise et al., 2017), and between the mPFC and insula (Kaiser et al., 2016), are all positively correlated with rumination scores in MDD; and that MDD patients generally spend longer time in the FC state associated with self-focused thinking during rest, which is related to their depressive severities and cognitive performances (Zhi et al., 2018).

MDD-related regional alterations were also found in several subcortical structures including basal-ganglia and thalamus, as well as sensorimotor cortical regions including the frontal and parietal areas (Fig. 3). A recent study in MDD reported increased temporal variability in basal-ganglia structures such as the pallidum, and suggested that it may be associated with impaired reward processing which could lead to anhedonia, one of the core clinical symptoms of MDD (Hou et al., 2018). Thus, our results further support this hypothesis. The thalamus and sensorimotor cortex are also known to be critical for relaying and processing sensory information in the brain (Brown et al., 2017; Cao et al., 2018). Therefore, excessive fluctuations of FC in these regions may reflect impairments in integrating information that underlies the emotional and sensory disturbances in MDD (Brown et al., 2017).

Our study has several limitations. First, some clinical information (e.g., dosage and duration of antidepressant treatment, treatment response, and use of mood stabilizers/antipsychotics) was not available in the current dataset, which has limited our abilities to further examine their possible effects. Second, the education levels were not matched between groups in the current sample. Although we have performed all the comparisons covarying for years of education and verified results in an education-matched subset, the results may partly be influenced by its effects. Third, the lengths of fMRI scans in this study were relatively short, which may to certain degree constrain the stability of the acquired signals. Lastly, while we proposed several clinical interpretations for all the observed changes in MDD patients, such as possible relationships with ruminations, they remain speculative and need to be tested in further studies.

## 5 Conclusions

In summary, we found that MDD is associated with increased temporal variability, decreased temporal clustering and increased temporal efficiency in dynamic functional brain networks during rest. These alterations mainly involved the default-mode, subcortical and sensorimotor regions, and were associated with depressive severity in patients, suggesting their important roles in the neuropathology of depression. Further studies are encouraged to replicate these findings and to examine their clinical associations with dysfunction in MDD patients in multiple domains.

## Declaration of Competing Interest

The authors report no biomedical financial interests or potential conflicts of interest related to the present work. Dr. Castellanos reports serving on the advisory board of BOL Pharma and receiving research support (study drug) from Greenwich Biosciences.

## Appendix Supplementary materials

Image, application 1
