# Supplementary material for: Altered resting-state dynamic functional brain networks in major depressive disorder: Findings from the REST-meta-MDD consortium
Source: Neuroimage Clin. 2020 Jan 7;26:102163. doi: 10.1016/j.nicl.2020.102163 (PMC7229351; doi:10.1016/j.nicl.2020.102163)

**SUPPLEMENTAL METHODS**

**Subjects**

The data in the present study was drawn from the REST-meta-MDD project, which has currently included 1300 patients with MDD and 1128 healthy subjects from 25 study sites across China (Yan et al., 2019). All participants are right-handed Han Chinese. Details about this project can be found elsewhere (Yan et al., 2019) and the dataset is publicly available at: http://rfmri.org/REST-meta-MDD.

The data acquisition parameters were varied across different sites in the REST-meta-MDD consortium. For example, the repetition time ranges from 2 to 3 seconds, and the number of time points ranges from 100 to 250 (Yan et al., 2019). To minimize the potential bias caused by varied parameters and to make sure that the fMRI scans are long enough for the analysis of dynamic FC, we selected the subjects using the following inclusion criteria: 1) 18–65 years of age; 2) repetition time = 2 seconds and the fMRI scan time ≥ 8 minutes (number of time points ≥ 240). There were 624 MDD patients and 515 HCs who met the above criteria. We further excluded 1) 85 MDD patients who were in clinical remission at the time of the scan (defined by HAMD scores ≤ 7); 2) 10 MDD patients and 5 HCs because of incomplete demographic information on education level; 3) 69 MDD patients and 37 HCs because of excessive head motion or poor data quality (see details in **Data Acquisition, Preprocessing and Quality Control**). Thereafter, the final analyzed sample consisted of 460 MDD patients and 473 HCs from 9 study sites.

Episodicity and medication information were available for a total of 372 patients from 6 sites, who were further divided into 155 FEDN and 217 non-FEDN patients based on their episode and medication statuses. The following subgroup analyses were performed among the three subgroups of FEDN (*N* = 155) and non-FEDN (*N* = 217) patients as well as HCs (*N* = 396) from these 6 sites.

**Data Acquisition, Preprocessing and Quality Control**

Resting-state fMRI and structural T1-weighted MRI brain scans were acquired at each site and were preprocessed locally using the DPARSF software (Yan, 2010) with a standardized protocol whose details can be found elsewhere (Yan et al., 2019). Briefly, it included removing the first 10 volumes for signal equilibrium, slice-timing correction, head motion realignment, brain tissue segmentation, spatial normalization and temporal filtering (0.01-0.10Hz). To control for head motion and physiological noises, the Friston-24 head motion parameters(Friston et al., 1996), liner trend, as well as signals from the white matter, cerebrospinal fluid and whole brain were regressed out. We performed global signal regression here, as recent studies have suggested that the temporal variability of FC is sensitive to artifacts induced by head motion (Laumann et al., 2017), and global signal regression is effective for mitigating this problem (Lydon-Staley et al., 2019). Additionally, the mean FD (Jenkinson et al., 2002) was calculated for excluding the subjects with excessive head motion and addressing the residual effects of head motion as a covariate in group analyses.

A total of 69 MDD patients and 37 HCs were excluded because of excessive head motion or poor data quality: 1) 31 MDD patients and 24 HCs were excluded because of poor imaging quality or inaccurate spatial normalization, determined by a careful manual checking; 2) 11 MDD patients and 9 HCs were excluded because of excessive head motion, determined by the mean FD > 0.2 mm; 3) 27 MDD patients and 4 HCs were excluded for bad coverage (fMRI signal was lost in any ROI of the Dosenbach/AAL atlas). The final analyzed sample consisted of 460 MDD patients and 473 HCs after excluding these subjects.

Of note, the lengths of preprocessed time series ranged from 460 to 480 seconds. To avoid the potential bias caused by different signal lengths, for all participants, only the signals of the first 460 seconds were extracted and fed into following analyses.

**Construction of Dynamic Brain Network**

To construct multi-layer dynamic networks, mean time series of each of the 160 ROIs in the Dosenbach functional atlas (Dosenbach et al., 2010) were firstly extracted from the 5-mm-radius spheres centered on previously reported coordinates as listed in **Supplemental Table S4**. A widely-used sliding-window approach (Laumann et al., 2017; Reinen et al., 2018; Sun et al., 2019) was then applied on the extracted time series, dividing them into a number of continuous time windows. According to the previous recommendations that the window length should exceed the inverse of the slowest frequency component in the signals (Leonardi and Van De Ville, 2015; Zalesky and Breakspear, 2015), a window length of 100 s and a validated incremental step of 6 s (Sun et al., 2019) were used in the primary analyses. This produced a total of 61 windows for each time series and the 160 × 160 whole-brain connectivity matrices were then calculated within each window using pairwise Pearson correlations, with all negative correlations in matrices retained here. As a result, a dynamic network *G* = (*G_t_*)*_t_* _= 1, 2, 3, …, 61_, where *G_t_* is the subgraph representing brain FC within the *t*th time window, was obtained for each subject.

**Dynamic Brain Network Metrics**

The metrics of temporal variability, temporal clustering and temporal efficiency were calculated as following:

**Temporal variability**: Following previous studies (Dong et al., 2019; Hou et al., 2018; Zhang et al., 2016), the temporal variability of a dynamic brain network was estimated by the average dissimilarity of its FC architectures between different time windows. The *nodal temporal variability* (*V_k_*) was firstly calculated to measure the regional variability of a ROI *k* as

$V_{k}=1-\overline{\mathrm{corrcoef}\left( F\left( i,k \right), F\left( j, k \right) \right)}$, *i*, *j* = 1, 2, 3, …, *T*; *i* ≠ *j*,

where *T* (= 61 here) is the total number of time windows, *F*(*i*, *k*) is the vector characterizing FC between ROI *k* and all other ROIs within the *i*th window (**Supplemental Fig. S1**), and “corrcoef” means correlation coefficients. *Temporal variability* (*V*) of the whole brain network was then calculated by averaging *V_k_* of all 160 ROIs as

$\text{V}=\frac{\sum_{\text{k}} \text{V}_{\text{k}}}{\text{N}}$,

where *N* is equal to 160 here. Both the obtained temporal variability and nodal temporal variability range from 0 to 2, and a higher value indicates a higher variability.

**Temporal clustering**: The temporal clustering is defined by the average topological overlap of each node's neighbors between any two consecutive time windows (Sizemore and Bassett, 2018; Tang et al., 2010). It was calculated at both global and regional levels, named as the *temporal correlation coefficient* and *nodal temporal correlation coefficient*, respectively. Let a*_ij_* (*t*) =1 when nodes *i* and *j* are connected within the *t*th time window and a*_ij_* (*t*) = 0 otherwise. The *nodal temporal correlation coefficient* of node *i* (*C_i_*) and *temporal correlation coefficient* of the whole network (*C*) were computed as (Sizemore and Bassett, 2018)

$C_{i}=\frac{1}{T-1}\sum_{t=1}^{T-1} \frac{\sum_{j} a_{ij}(t)a_{ij}(t+1)}{\sqrt{\left[ \sum_{j} a_{ij}(t) \right]\left[ \sum_{j} a_{ij}(t+1) \right]}}$ and $\text{C}=\frac{\sum_{\text{i}} \text{C}_{\text{i}}}{\text{N}}$,

where *T* (= 61 here) and *N* (= 160 here) are the number of windows and nodes, respectively. Both *C_i_* and *C* are in the range of [0, 1], with a higher value indicating a higher overlapping of each node’s neighbors between consecutive time windows (a higher temporal clustering) (Sizemore and Bassett, 2018); in particular, *C* = 1 when all nodes’ neighbors never change over time (see an example in **Supplemental Fig. S2A**).

**Temporal efficiency**: The temporal efficiency quantifies how quickly information can be transmitted between nodes in a dynamic network (Sizemore and Bassett, 2018; Thompson et al., 2017). In a dynamic network, even if the nodes *i* and *j* are not directly connected within any time window, information can still be transferred from node *i* can to node *j* along a path *i* → *k*_1_ → *k*_2_ → … → *k_n_*_-1_ → *j* when there is a time-ordered sequence of connections that

$a_{ik_{1}}\left( t_{1} \right)=a_{k_{1}k_{2}}\left( t_{2} \right)=a_{k_{2}k_{3}}\left( t_{3} \right)=\ldots=a_{k_{n-1}j}\left( t_{n} \right)=1$,

where 1 ≤ *t*_1_ < *t*_2_ < …< *t_n_* ≤ *T* (Sizemore and Bassett, 2018). Such a path is called a *temporal path* whose length is (*t_n_* - *t*_1_ + 1) (Thompson et al., 2017). The length of the shortest temporal path from nodes *i* to *j* is called *temporal distance* *d_ij_* ranging from 1 to infinite (**Supplemental Fig. S2B**). Note that the temporal distance between the directly-connected nodes is 1, and that *d_ij_* does not necessarily equate to *d_ji_*. Then we computed the *nodal* *temporal path length* of node *i* (*L_i_*) and *characteristic temporal path length* of the whole network (*L*) as (Sizemore and Bassett, 2018)

$L_{i}=\frac{2(N-1)}{\sum_{j} (\frac{1}{d_{ij}}+\frac{1}{d_{ji}})}$ and $L=\frac{N(N-1)}{\sum_{i\neq j} \frac{1}{d_{ij}}}$,

where the harmonic mean was used here to handle infinite values. A shorter *L_i_ or L* indicates a shorter average temporal distance between nodes or in other word, that information can be transferred between nodes in a shorter time on average (a higher temporal efficiency).

Note that temporal clustering and temporal efficiency are only defined for binary networks in current (Sizemore and Bassett, 2018) and for their calculations, we obtained binary dynamic networks by preserving only a particular proportion (“sparsity”) of the strongest connections between nodes on the FC matrices of each window. Here, we applied a range of sparsities from 10% to 50% with an increment interval of 1%, in which graph metrics could be estimated properly and the number of spurious edges would be minimized (Sreenivasan et al., 2017; Zhang et al., 2019, 2011). At each sparsity level, edges in the networks were defined by assigning a value of 1 to the connections who survived the given threshold and a value of 0 to those who did not. As a result, a dynamic network *G* = (*G_t_*)*_t_* _= 1, 2, 3, …, 61_, where *G_t_* is the binary subgraph representing brain FC within the *t*th time window, was obtained for each subject at each sparsity level. Metrics of the temporal clustering and temporal efficiency were then computed at each sparsity level separately.

The metrics of temporal clustering and temporal efficiency were computed by a publicly-available MATLAB toolbox (https://github.com/asizemore/Dynamic-Graph-Metrics), whose details can be found in a previous publication (Sizemore and Bassett, 2018).

**Validation Analyses**

**Impact of parcellation schemes**: To validate that our results were not biased by the preselected parcellation scheme, we repeated the group comparisons on all metrics using the AAL atlas (Tzourio-Mazoyer et al., 2002) with 90 ROIs at both global and regional levels.

**Sliding-window lengths**: To estimate the reproducibility of results across different analysis parameters, we repeated the group comparisons on all metrics using a set of different window and step lengths (window/step = [40, 60, 80, 100]/[4, 6, 8] seconds) in constructing dynamic networks.

**Subset analyses**: To evaluate whether the results were affected by sample population or unmatched education levels, group differences on all metrics were tested within each of the following subsets: 1) the subsets of each individual site; 2) two split-half subsets randomly split from the whole sample. Randomization was achieved as follows: a random number within the interval [0, 1] was firstly generated for each subject; after that, the subjects with a number < 0.5 and >= 0.5 were assigned to the two subsets, respectively; and 3) a subset extracted from the whole sample where education levels were matched between groups, by excluding all healthy subjects with years of education ≥ 16 or age ≥ 60.

**SUPPLEMENTAL REFERENCES**

Dong, D., Duan, M., Wang, Y., Zhang, X., Jia, X., Li, Y., Xin, F., Yao, D., Luo, C., 2019. Reconfiguration of Dynamic Functional Connectivity in Sensory and Perceptual System in Schizophrenia. Cereb. Cortex. https://doi.org/10.1093/cercor/bhy232

Dosenbach, N.U.F., Nardos, B., Cohen, A.L., Fair, D.A., Power, J.D., Church, J.A., Nelson, S.M., Wig, G.S., Vogel, A.C., Lessov-Schlaggar, C.N., Barnes, K.A., Dubis, J.W., Feczko, E., Coalson, R.S., Pruett, J.R., Barch, D.M., Petersen, S.E., Schlaggar, B.L., 2010. Prediction of individual brain maturity using fMRI. Science (80-. ). https://doi.org/10.1126/science.1194144

Friston, K.J., Williams, S., Howard, R., Frackowiak, R.S.J., Turner, R., 1996. Movement-related effects in fMRI time-series. Magn. Reson. Med. https://doi.org/10.1002/mrm.1910350312

Hou, Z., Kong, Y., He, X., Yin, Y., Zhang, Y., Yuan, Y., 2018. Increased temporal variability of striatum region facilitating the early antidepressant response in patients with major depressive disorder. Prog. Neuro-Psychopharmacology Biol. Psychiatry. https://doi.org/10.1016/j.pnpbp.2018.03.026

Jenkinson, M., Bannister, P., Brady, M., Smith, S., 2002. Improved optimization for the robust and accurate linear registration and motion correction of brain images. Neuroimage. https://doi.org/10.1016/S1053-8119(02)91132-8

Laumann, T.O., Snyder, A.Z., Mitra, A., Gordon, E.M., Gratton, C., Adeyemo, B., Gilmore, A.W., Nelson, S.M., Berg, J.J., Greene, D.J., McCarthy, J.E., Tagliazucchi, E., Laufs, H., Schlaggar, B.L., Dosenbach, N.U.F., Petersen, S.E., 2017. On the Stability of BOLD fMRI Correlations. Cereb. Cortex. https://doi.org/10.1093/cercor/bhw265

Leonardi, N., Van De Ville, D., 2015. On spurious and real fluctuations of dynamic functional connectivity during rest. Neuroimage. https://doi.org/10.1016/j.neuroimage.2014.09.007

Lydon-Staley, D.M., Ciric, R., Satterthwaite, T.D., Bassett, D.S., 2019. Evaluation of confound regression strategies for the mitigation of micromovement artifact in studies of dynamic resting-state functional connectivity and multilayer network modularity. Netw. Neurosci. https://doi.org/10.1162/netn_a_00071

Reinen, J.M., Chén, O.Y., Hutchison, R.M., Yeo, B.T.T., Anderson, K.M., Sabuncu, M.R., Öngür, D., Roffman, J.L., Smoller, J.W., Baker, J.T., Holmes, A.J., 2018. The human cortex possesses a reconfigurable dynamic network architecture that is disrupted in psychosis. Nat. Commun. https://doi.org/10.1038/s41467-018-03462-y

Sizemore, A.E., Bassett, D.S., 2018. Dynamic graph metrics: Tutorial, toolbox, and tale. Neuroimage. https://doi.org/10.1016/j.neuroimage.2017.06.081

Sreenivasan, K., Zhuang, X., Banks, S.J., Mishra, V., Yang, Z., Deshpande, G., Cordes, D., 2017. Olfactory Network Differences in Master Sommeliers: Connectivity Analysis Using Granger Causality and Graph Theoretical Approach. Brain Connect. https://doi.org/10.1089/brain.2016.0458

Sun, Y., Collinson, S.L., Suckling, J., Sim, K., 2019. Dynamic Reorganization of Functional Connectivity Reveals Abnormal Temporal Efficiency in Schizophrenia. Schizophr. Bull. https://doi.org/10.1093/schbul/sby077

Tang, J., Scellato, S., Musolesi, M., Mascolo, C., Latora, V., 2010. Small-world behavior in time-varying graphs. Phys. Rev. E - Stat. Nonlinear, Soft Matter Phys. https://doi.org/10.1103/PhysRevE.81.055101

Thompson, W.H., Brantefors, P., Fransson, P., 2017. From static to temporal network theory: Applications to functional brain connectivity. Netw. Neurosci. https://doi.org/10.1162/netn_a_00011

Tzourio-Mazoyer, N., Landeau, B., Papathanassiou, D., Crivello, F., Etard, O., Delcroix, N., Mazoyer, B., Joliot, M., 2002. Automated anatomical labeling of activations in SPM using a macroscopic anatomical parcellation of the MNI MRI single-subject brain. Neuroimage. https://doi.org/10.1006/nimg.2001.0978

Yan, 2010. DPARSF: a MATLAB toolbox for “pipeline” data analysis of resting-state fMRI. Front. Syst. Neurosci. https://doi.org/10.3389/fnsys.2010.00013

Yan, C.G., Chen, X., Li, L., Castellanos, F.X., Bai, T.J., Bo, Q.J., Cao, J., Chen, G.M., Chen, N.X., Chen, W., Cheng, C., Cheng, Y.Q., Cui, X.L., Duan, J., Fang, Y.R., Gong, Q.Y., Guo, W. Bin, Hou, Z.H., Hu, L., Kuang, L., Li, F., Li, K.M., Li, T., Liu, Y.S., Liu, Z.N., Long, Y.C., Luo, Q.H., Meng, H.Q., Peng, D.H., Qiu, H.T., Qiu, J., Shen, Y. Di, Shi, Y.S., Wang, C.Y., Wang, F., Wang, K., Wang, L., Wang, X., Wang, Y., Wu, X.P., Wu, X.R., Xie, C.M., Xie, G.R., Xie, H.Y., Xie, P., Xu, X.F., Yang, H., Yang, J., Yao, J.S., Yao, S.Q., Yin, Y.Y., Yuan, Y.G., Zhang, A.X., Zhang, H., Zhang, K.R., Zhang, L., Zhang, Z.J., Zhou, R.B., Zhou, Y.T., Zhu, J.J., Zou, C.J., Si, T.M., Zuo, X.N., Zhao, J.P., Zang, Y.F., 2019. Reduced default mode network functional connectivity in patients with recurrent major depressive disorder. Proc. Natl. Acad. Sci. U. S. A. https://doi.org/10.1073/pnas.1900390116

Zalesky, A., Breakspear, M., 2015. Towards a statistical test for functional connectivity dynamics. Neuroimage. https://doi.org/10.1016/j.neuroimage.2015.03.047

Zhang, J., Cheng, W., Liu, Z., Zhang, K., Lei, X., Yao, Y., Becker, B., Liu, Y., Kendrick, K.M., Lu, G., Feng, J., 2016. Neural, electrophysiological and anatomical basis of brain-network variability and its characteristic changes in mental disorders. Brain. https://doi.org/10.1093/brain/aww143

Zhang, M., Wang, S., Hu, D., Kang, H., Ouyang, M., Zhang, Y., Rao, B., Huang, H., Peng, Y., 2019. Altered brain functional network in children with type 1 Gaucher disease: a longitudinal graph theory-based study. Neuroradiology. https://doi.org/10.1007/s00234-018-2104-3

Zhang, T., Wang, J., Yang, Y., Wu, Q., Li, B., Chen, L., Yue, Q., Tang, H., Yan, C., Lui, S., Huang, X., Chan, R.C.K., Zang, Y., He, Y., Gong, Q., 2011. Abnormal small-world architecture of top-down control networks in obsessive-compulsive disorder. J. Psychiatry Neurosci. https://doi.org/10.1503/jpn.100006

**Supplemental Table S1.** The published studies which have investigated temporal variability of resting-state FC in MDD.

| Authors | Sample size | Major findings in MDD patients |
| --- | --- | --- |
| Kaiser et al. [31] | 100 MDD patients and 109 HCs | Increased temporal variability of FC between mPFC and insula; decreased temporal variability of FC between mPFC and parahippocampal gyrus |
| Wise et al. [32] | Two samples: 20 MDD patients and 19 HCs/19 MDD patients and 19 HCs | Increased temporal variability of FC between mPFC and posterior cingulate cortex, which were replicated across the two independent samples |
| Demirtaş et al. [33] | 27 MDD patients/27 HCs | Decreased temporal variability of FC at the global level, as well as between several components of the default-mode and frontoparietal networks |
| Hou et al. [10] | 40 antidepressant-responsive (RD) MDD patients, 37 non-responding (NRD) patients, and 42 HCs | Increased temporal variability of FC in the inferior frontal gyrus (both RD and NRD patients) and pallidum (only RD patients) |

Abbreviations: MDD = major depressive disorder; HCs = healthy controls; FC = functional connectivity; mPFC = medial prefrontal cortex.

**Supplemental Table S2.** The 160 ROIs used to define network nodes with the Montreal Neurological Institute (MNI) coordinates, which were taken from (Dosenbach et al., 2010).

| Node index | MNI x | MNI y | MNI z | Label | Node index | MNI x | MNI y | MNI z | Label |
| --- | --- | --- | --- | --- | --- | --- | --- | --- | --- |
| 1 | 6 | 64 | 3 | vmPFC | 81 | 54 | -31 | -18 | fusiform |
| 2 | 29 | 57 | 18 | aPFC | 82 | -41 | -37 | 16 | temporal |
| 3 | -29 | 57 | 10 | aPFC | 83 | -53 | -37 | 13 | temporal |
| 4 | 0 | 51 | 32 | mPFC | 84 | 28 | -37 | -15 | fusiform |
| 5 | -25 | 51 | 27 | aPFC | 85 | -3 | -38 | 45 | precuneus |
| 6 | 9 | 51 | 16 | vmPFC | 86 | 34 | -39 | 65 | sup-parietal |
| 7 | -6 | 50 | -1 | vmPFC | 87 | 8 | -40 | 50 | precuneus |
| 8 | 27 | 49 | 26 | aPFC | 88 | -41 | -40 | 42 | IPL |
| 9 | 42 | 48 | -3 | vent-aPFC | 89 | 58 | -41 | 20 | parietal |
| 10 | -43 | 47 | 2 | vent-aPFC | 90 | -8 | -41 | 3 | post-cingulate |
| 11 | -11 | 45 | 17 | vmPFC | 91 | -61 | -41 | -2 | inf-temporal |
| 12 | 39 | 42 | 16 | vlPFC | 92 | -28 | -42 | -11 | occipital |
| 13 | 8 | 42 | -5 | vmPFC | 93 | -5 | -43 | 25 | post-cingulate |
| 14 | 9 | 39 | 20 | ACC | 94 | 9 | -43 | 25 | precuneus |
| 15 | 46 | 39 | -15 | vlPFC | 95 | 43 | -43 | 8 | temporal |
| 16 | 40 | 36 | 29 | dlPFC | 96 | 54 | -44 | 43 | IPL |
| 17 | 23 | 33 | 47 | sup-frontal | 97 | -55 | -44 | 30 | parietal |
| 18 | 34 | 32 | 7 | vPFC | 98 | -28 | -44 | -25 | lat-cerebellum |
| 19 | -2 | 30 | 27 | ACC | 99 | -35 | -46 | 48 | post-parietal |
| 20 | -16 | 29 | 54 | sup-frontal | 100 | 42 | -46 | 21 | sup-temporal |
| 21 | -1 | 28 | 40 | ACC | 101 | -48 | -47 | 49 | IPL |
| 22 | 46 | 28 | 31 | dlPFC | 102 | -41 | -47 | 29 | angular-gyrus |
| 23 | -52 | 28 | 17 | vPFC | 103 | -59 | -47 | 11 | temporal |
| 24 | -44 | 27 | 33 | dlPFC | 104 | -53 | -50 | 39 | IPL |
| 25 | 51 | 23 | 8 | vFC | 105 | 5 | -50 | 33 | precuneus |
| 26 | 38 | 21 | -1 | ant-insula | 106 | -18 | -50 | 1 | occipital |
| 27 | 9 | 20 | 34 | dACC | 107 | 44 | -52 | 47 | IPL |
| 28 | -36 | 18 | 2 | ant-insula | 108 | -5 | -52 | 17 | post-cingulate |
| 29 | 40 | 17 | 40 | dFC | 109 | -24 | -54 | -21 | lat-cerebellum |
| 30 | -6 | 17 | 34 | basal-ganglia | 110 | -37 | -54 | -37 | inf-cerebellum |
| 31 | 0 | 15 | 45 | mFC | 111 | 10 | -55 | 17 | post-cingulate |
| 32 | 58 | 11 | 14 | frontal | 112 | -6 | -56 | 29 | precuneus |
| 33 | -46 | 10 | 14 | vFC | 113 | -34 | -57 | -24 | lat-cerebellum |
| 34 | 44 | 8 | 34 | dFC | 114 | -32 | -58 | 46 | IPS |
| 35 | 60 | 8 | 34 | dFC | 115 | -11 | -58 | 17 | post-cingulate |
| 36 | -42 | 7 | 36 | dFC | 116 | 32 | -59 | 41 | IPS |
| 37 | -55 | 7 | 23 | vFC | 117 | 51 | -59 | 34 | angular-gyrus |
| 38 | -20 | 6 | 7 | basal-ganglia | 118 | -34 | -60 | -5 | occipital |
| 39 | 14 | 6 | 7 | basal-ganglia | 119 | 36 | -60 | -8 | occipital |
| 40 | -48 | 6 | 1 | vFC | 120 | -6 | -60 | -15 | med-cerebellum |
| 41 | 10 | 5 | 51 | pre-SMA | 121 | -25 | -60 | -34 | inf-cerebellum |
| 42 | 43 | 1 | 12 | vFC | 122 | 32 | -61 | -31 | inf-cerebellum |
| 43 | 0 | -1 | 52 | SMA | 123 | 46 | -62 | 5 | temporal |
| 44 | 37 | -2 | -3 | mid-insula | 124 | -48 | -63 | 35 | angular-gyrus |
| 45 | 53 | -3 | 32 | frontal | 125 | -52 | -63 | 15 | TPJ |
| 46 | 58 | -3 | 17 | precentral-gyrus | 126 | -44 | -63 | -7 | occipital |
| 47 | -12 | -3 | 13 | thalamus | 127 | -16 | -64 | -21 | med-cerebellum |
| 48 | -42 | -3 | 11 | mid-insula | 128 | 21 | -64 | -22 | lat-cerebellum |
| 49 | -44 | -6 | 49 | precentral-gyrus | 129 | 19 | -66 | -1 | occipital |
| 50 | -26 | -8 | 54 | parietal | 130 | 1 | -66 | -24 | med-cerebellum |
| 51 | 46 | -8 | 24 | precentral-gyrus | 131 | -34 | -67 | -29 | inf-cerebellum |
| 52 | -54 | -9 | 23 | precentral-gyrus | 132 | 11 | -68 | 42 | precuneus |
| 53 | 44 | -11 | 38 | precentral-gyrus | 133 | 17 | -68 | 20 | occipital |
| 54 | -47 | -12 | 36 | parietal | 134 | -36 | -69 | 40 | IPS |
| 55 | 33 | -12 | 16 | mid-insula | 135 | 39 | -71 | 13 | occipital |
| 56 | -36 | -12 | 15 | mid-insula | 136 | -9 | -72 | 41 | occipital |
| 57 | -12 | -12 | 6 | thalamus | 137 | 45 | -72 | 29 | occipital |
| 58 | 11 | -12 | 6 | thalamus | 138 | -11 | -72 | -14 | med-cerebellum |
| 59 | 32 | -12 | 2 | mid-insula | 139 | 29 | -73 | 29 | occipital |
| 60 | 59 | -13 | 8 | temporal | 140 | 33 | -73 | -30 | inf-cerebellum |
| 61 | -30 | -14 | 1 | mid-insula | 141 | -2 | -75 | 32 | occipital |
| 62 | -38 | -15 | 59 | parietal | 142 | -29 | -75 | 28 | occipital |
| 63 | 52 | -15 | -13 | inf-temporal | 143 | 5 | -75 | -11 | med-cerebellum |
| 64 | -47 | -18 | 50 | parietal | 144 | 14 | -75 | -21 | med-cerebellum |
| 65 | 46 | -20 | 45 | parietal | 145 | -16 | -76 | 33 | occipital |
| 66 | -55 | -22 | 38 | parietal | 146 | -42 | -76 | 26 | occipital |
| 67 | -54 | -22 | 22 | precentral-gyrus | 147 | 9 | -76 | 14 | occipital |
| 68 | -54 | -22 | 9 | temporal | 148 | 15 | -77 | 32 | occipital |
| 69 | 41 | -23 | 55 | parietal | 149 | 20 | -78 | -2 | occipital |
| 70 | 42 | -24 | 17 | post-insula | 150 | -21 | -79 | -33 | inf-cerebellum |
| 71 | 11 | -24 | 2 | basal-ganglia | 151 | -6 | -79 | -33 | inf-cerebellum |
| 72 | -59 | -25 | -15 | inf-temporal | 152 | -5 | -80 | 9 | post-occipital |
| 73 | 1 | -26 | 31 | post-cingulate | 153 | 29 | -81 | 14 | post-occipital |
| 74 | 18 | -27 | 62 | parietal | 154 | 33 | -81 | -2 | post-occipital |
| 75 | -38 | -27 | 60 | parietal | 155 | 18 | -81 | -33 | inf-cerebellum |
| 76 | -30 | -28 | 9 | post-insula | 156 | -37 | -83 | -2 | post-occipital |
| 77 | -24 | -30 | 64 | parietal | 157 | -29 | -88 | 8 | post-occipital |
| 78 | 51 | -30 | 5 | temporal | 158 | 13 | -91 | 2 | post-occipital |
| 79 | -41 | -31 | 48 | post-parietal | 159 | 27 | -91 | 2 | post-occipital |
| 80 | -4 | -31 | -4 | post-cingulate | 160 | -4 | -94 | 12 | post-occipital |

**Supplemental Table S3.** The demographic, clinical and image (head motion) characteristics of the subgroups.

|  | FEDN (*n* = 155) | Non-FEDN (*n* = 217) | Healthy controls (*n* = 396) | Comparisons |
| --- | --- | --- | --- | --- |
|  | (Mean ± SD) | (Mean ± SD) | (Mean ± SD) |  |
| Age, years | 38.200 ± 12.598 | 36.912 ±12.273 | 38.200 ±14.540 | *F* = 0.702, *p* = 0.496 |
| Sex, male/female | 46/109 | 80/137 | 146/250 | *χ*^2^ = 2.796, *p* = 0.247 |
| Education level, years | 11.471 ± 3.493 | 11.134 ± 3.227 | 12.116 ± 3.614 | *F* = 21.026, *p* < 0.001*^a^* |
| Mean FD | 0.067 ± 0.030 | 0.070 ± 0.034 | 0.073 ± 0.035 | *F* = 1.936, *p* = 0.145 |
| 17-item HAMD scores | 24.342 ± 5.946 | 20.313 ± 6.197 | / | *t* = 6.286, *p* < 0.001 |
| Duration of illness, months*^b^* | 26.517 ± 41.495 | 76.162 ± 85.548 | / | *t* = 7.288, *p* < 0.001 |

Abbreviations: FEDN = first-episode and drug-naïve patients; SD = standard deviation; FD = framewise-displacement; HAMD = Hamilton Depression Rating Scale.

*^a^*The LSD post-hoc tests showed that both the FEDN and non-FEDN patients have a significantly lower education level than healthy controls, while no significant difference was found between the FEDN and non-FEDN subgroups.

*^b^*Data on the duration of illness was available for 145 FEDN and 212 non-FEDN patients.

**Supplemental Table S4.** The demographic, clinical and image (head motion) characteristics of the groups in each subset.

|  |  | Major depressive disorder | Health controls | Group comparisons |
| --- | --- | --- | --- | --- |
|  |  | (Mean ± SD) | (Mean ± SD) |  |
| Split-half subset 1 | Number | 239 | 244 | / |
|  | Age, years | 35.891 ± 13.037 | 37.225 ±15.635 | *t* = -1.019, *p* = 0.309 |
|  | Sex, male/female | 78/161 | 91/153 | *χ*^2^ = 1.152, *p* = 0.283 |
|  | Education level, years | 11.431 ± 3.210 | 12.910 ± 3.373 | *t* = -4.936, *p* < 0.001 |
|  | Mean FD | 0.066 ± 0.032 | 0.070 ± 0.036 | *t* = -1.094, *p* = 0.274 |
| Split-half subset 2 | Number | 221 | 229 | */* |
|  | Age, years | 37.751 ± 13.366 | 36.576 ±14.859 | *t* = 0.882, *p* = 0.379 |
|  | Sex, male/female | 77/144 | 86/143 | *χ*^2^ = 0.358, *p* = 0.549 |
|  | Education level, years | 11.423 ± 3.268 | 13.053 ± 3.594 | *t* = -5.028, *p* < 0.001 |
|  | Mean FD | 0.070 ± 0.034 | 0.070 ± 0.035 | *t* = -0.109, *p* = 0.913 |
| Education-matched subset | Number | 460 | 283 | / |
|  | Age, years | 36.785 ± 13.215 | 37.675 ±13.696 | *t* = -0.879, *p* = 0.255 |
|  | Sex, male/female | 155/305 | 107/176 | *χ*^2^ = 1.299, *p* = 0.254 |
|  | Education level, years | 11.427 ± 3.235 | 11.513 ± 2.813 | *t* = -0.383, *p* = 0.702 |
|  | Mean FD | 0.068 ± 0.033 | 0.069 ± 0.035 | *t* = -0.643, *p* = 0.520 |

Abbreviations: SD = standard deviation; FD = framewise-displacement; HAMD = Hamilton Depression Rating Scale.

**Supplemental Table S5.** Comparisons of the temporal correlation coefficient and characteristic temporal path length at each sparsity level.

| Sparsity of (%) | Temporal correlation coefficient | | | Characteristic temporal path length | | |
| --- | --- | --- | --- | --- | --- | --- |
|  | Comparisons | *F* | *p* | Comparisons | *F* | *p* |
| 10 | MDD < HCs | 13.301 | 2.80×10^-4^ | MDD < HCs | 10.294 | 0.001 |
| 11 | MDD < HCs | 13.087 | 3.14×10^-4^ | MDD < HCs | 10.446 | 0.001 |
| 12 | MDD < HCs | 12.791 | 3.66×10^-4^ | MDD < HCs | 10.774 | 0.001 |
| 13 | MDD < HCs | 13.240 | 2.89×10^-4^ | MDD < HCs | 10.906 | 0.001 |
| 14 | MDD < HCs | 15.184 | 1.05×10^-4^ | MDD < HCs | 10.452 | 0.001 |
| 15 | MDD < HCs | 15.838 | 7.44×10^-5^ | MDD < HCs | 10.761 | 0.001 |
| 16 | MDD < HCs | 14.376 | 1.59×10^-4^ | MDD < HCs | 10.264 | 0.001 |
| 17 | MDD < HCs | 15.128 | 1.08×10^-4^ | MDD < HCs | 9.925 | 0.002 |
| 18 | MDD < HCs | 15.387 | 9.41×10^-5^ | MDD < HCs | 9.981 | 0.002 |
| 19 | MDD < HCs | 14.951 | 1.18×10^-4^ | MDD < HCs | 9.479 | 0.002 |
| 20 | MDD < HCs | 15.203 | 1.04×10^-4^ | MDD < HCs | 9.550 | 0.002 |
| 21 | MDD < HCs | 14.155 | 1.79×10^-4^ | MDD < HCs | 9.345 | 0.002 |
| 22 | MDD < HCs | 14.672 | 1.37×10^-4^ | MDD < HCs | 9.664 | 0.002 |
| 23 | MDD < HCs | 14.918 | 1.20×10^-4^ | MDD < HCs | 9.613 | 0.002 |
| 24 | MDD < HCs | 14.390 | 1.58×10^-4^ | MDD < HCs | 9.381 | 0.002 |
| 25 | MDD < HCs | 12.962 | 3.35×10^-4^ | MDD < HCs | 9.019 | 0.003 |
| 26 | MDD < HCs | 12.593 | 4.07×10^-4^ | MDD < HCs | 8.934 | 0.003 |
| 27 | MDD < HCs | 13.406 | 2.65×10^-4^ | MDD < HCs | 8.882 | 0.003 |
| 28 | MDD < HCs | 13.113 | 3.09×10^-4^ | MDD < HCs | 8.649 | 0.003 |
| 29 | MDD < HCs | 13.328 | 2.76×10^-4^ | MDD < HCs | 8.377 | 0.004 |
| 30 | MDD < HCs | 13.611 | 2.38×10^-4^ | MDD < HCs | 8.461 | 0.004 |
| 31 | MDD < HCs | 13.708 | 2.26×10^-4^ | MDD < HCs | 7.991 | 0.005 |
| 32 | MDD < HCs | 13.719 | 2.25×10^-4^ | MDD < HCs | 8.163 | 0.004 |
| 33 | MDD < HCs | 14.150 | 1.79×10^-4^ | MDD < HCs | 7.735 | 0.006 |
| 34 | MDD < HCs | 14.840 | 1.25×10^-4^ | MDD < HCs | 7.319 | 0.007 |
| 35 | MDD < HCs | 14.590 | 1.43×10^-4^ | MDD < HCs | 7.345 | 0.007 |
| 36 | MDD < HCs | 13.885 | 2.06×10^-4^ | MDD < HCs | 7.068 | 0.008 |
| 37 | MDD < HCs | 13.790 | 2.17×10^-4^ | MDD < HCs | 6.655 | 0.010 |
| 38 | MDD < HCs | 13.713 | 2.26×10^-4^ | MDD < HCs | 6.410 | 0.012 |
| 39 | MDD < HCs | 13.354 | 2.73×10^-4^ | MDD < HCs | 6.361 | 0.012 |
| 40 | MDD < HCs | 13.790 | 2.17×10^-4^ | MDD < HCs | 6.467 | 0.011 |
| 41 | MDD < HCs | 13.877 | 2.07×10^-4^ | MDD < HCs | 6.296 | 0.012 |
| 42 | MDD < HCs | 14.158 | 1.79×10^-4^ | MDD < HCs | 6.033 | 0.014 |
| 43 | MDD < HCs | 14.069 | 1.87×10^-4^ | MDD < HCs | 5.631 | 0.018 |
| 44 | MDD < HCs | 14.115 | 1.83×10^-4^ | MDD < HCs | 5.633 | 0.018 |
| 45 | MDD < HCs | 14.529 | 1.47×10^-4^ | MDD < HCs | 5.686 | 0.017 |
| 46 | MDD < HCs | 15.177 | 1.05×10^-4^ | MDD < HCs | 5.560 | 0.019 |
| 47 | MDD < HCs | 15.014 | 1.14×10^-4^ | MDD < HCs | 5.205 | 0.023 |
| 48 | MDD < HCs | 14.756 | 1.31×10^-4^ | MDD < HCs | 4.959 | 0.026 |
| 49 | MDD < HCs | 14.526 | 1.47×10^-4^ | MDD < HCs | 4.916 | 0.027 |
| 50 | MDD < HCs | 14.520 | 1.48×10^-4^ | MDD < HCs | 4.594 | 0.032 |

Abbreviations: MDD = major depressive disorder; HCs = healthy controls.

**Supplemental Table S6.** The ROIs with significant between-group differences (FDR-corrected *p* <0.05) in nodal temporal variability, nodal temporal correlation coefficient and nodal temporal path length.

| MNI x | MNI y | MNI z | Label | Nodal temporal variability | | Nodal temporal correlation coefficient | | Nodal temporal path length | |
| --- | --- | --- | --- | --- | --- | --- | --- | --- | --- |
|  |  |  |  | Comparisons | FDR-corrected *p* | Comparisons | FDR-corrected *p* | Comparisons | FDR-corrected *p* |
| -6 | 50 | -1 | vmPFC |  |  |  |  | MDD < HCs | 0.048 |
| -11 | 45 | 17 | vmPFC |  |  |  |  | MDD < HCs | 0.047 |
| -2 | 30 | 27 | ACC |  |  |  |  | MDD < HCs | 0.048 |
| 44 | 8 | 34 | dFC |  |  | MDD < HCs | 0.042 | MDD < HCs | 0.048 |
| 60 | 8 | 34 | dFC | MDD > HCs | 0.048 |  |  | MDD < HCs | 0.004 |
| -20 | 6 | 7 | basal-ganglia |  |  |  |  | MDD < HCs | 0.010 |
| 14 | 6 | 7 | basal-ganglia |  |  |  |  | MDD < HCs | 0.017 |
| 53 | -3 | 32 | frontal |  |  | MDD < HCs | 0.030 |  |  |
| -26 | -8 | 54 | parietal | MDD > HCs | 0.048 |  |  | MDD < HCs | 0.010 |
| -54 | -9 | 23 | precentral-gyrus | MDD > HCs | 0.014 | MDD < HCs | 0.004 | MDD < HCs | 0.024 |
| -47 | -12 | 36 | parietal |  |  | MDD < HCs | 0.042 |  |  |
| -12 | -12 | 6 | thalamus | MDD > HCs | 0.031 |  |  | MDD < HCs | 0.042 |
| 11 | -12 | 6 | thalamus | MDD > HCs | 0.019 |  |  | MDD < HCs | 0.028 |
| -47 | -18 | 50 | parietal | MDD > HCs | 0.013 | MDD < HCs | 0.004 | MDD < HCs | 0.010 |
| 46 | -20 | 45 | parietal | MDD > HCs | 0.048 |  |  |  |  |
| -55 | -22 | 38 | parietal |  |  |  |  | MDD < HCs | 0.028 |
| 41 | -23 | 55 | parietal | MDD > HCs | 0.014 | MDD < HCs | 0.042 | MDD < HCs | 0.010 |
| -59 | -25 | -15 | inf-temporal | MDD > HCs | 0.043 |  |  | MDD < HCs | 0.009 |
| 9 | -43 | 25 | precuneus |  |  |  |  | MDD < HCs | 0.048 |
| -41 | -47 | 29 | angular-gyrus |  |  |  |  | MDD < HCs | 0.041 |
| 5 | -50 | 33 | precuneus | MDD > HCs | 0.048 |  |  |  |  |
| 10 | -55 | 17 | post-cingulate |  |  | MDD < HCs | 0.042 |  |  |
| -6 | -56 | 29 | precuneus | MDD > HCs | 0.048 |  |  | MDD < HCs | 0.020 |
| 51 | -59 | 34 | angular-gyrus |  |  | MDD < HCs | 0.048 |  |  |
| -48 | -63 | 35 | angular-gyrus |  |  |  |  | MDD < HCs | 0.028 |
| -36 | -69 | 40 | IPS | MDD > HCs | 0.048 |  |  | MDD < HCs | 0.018 |
| 29 | -73 | 29 | occipital | MDD > HCs | 0.031 | MDD < HCs | 0.042 | MDD < HCs | 0.045 |
| -37 | -83 | -2 | post-occipital |  |  |  |  | MDD < HCs | 0.028 |
| -29 | -88 | 8 | post-occipital | MDD > HCs | 0.048 |  |  |  |  |

Abbreviations: ACC = anterior cingulate cortex; dFC = dorsal frontal cortex; HCs = healthy controls; IPS = intraparietal sulcus; MDD = major depressive disorder; vmPFC = ventromedial prefrontal cortex.

**Supplemental Table S7.** Results of group comparisons on temporal variability, temporal correlation coefficient and characteristic temporal path length with different window and step lengths used in constructing dynamic networks.

| Window length /seconds | Step length /seconds | Temporal variability | | | Temporal correlation coefficient | | | Characteristic temporal path length | | |
| --- | --- | --- | --- | --- | --- | --- | --- | --- | --- | --- |
|  |  | Comparisons | *F* | *p* | Comparisons | *F* | *p* | Comparisons | *F* | *p* |
| 40 | 4 | MDD > HCs | 10.391 | 1.31×10^-3^ | MDD < HCs | 9.870 | 1.73×10^-3^ | MDD < HCs | 6.257 | 0.013 |
| 40 | 6 | MDD > HCs | 10.263 | 1.40×10^-3^ | MDD < HCs | 9.168 | 2.53×10^-3^ | MDD < HCs | 6.844 | 0.009 |
| 40 | 8 | MDD > HCs | 10.400 | 1.30×10^-3^ | MDD < HCs | 9.431 | 2.20×10^-3^ | MDD < HCs | 6.901 | 0.009 |
| 60 | 4 | MDD > HCs | 10.112 | 1.52×10^-3^ | MDD < HCs | 12.278 | 4.81×10^-4^ | MDD < HCs | 7.398 | 0.007 |
| 60 | 6 | MDD > HCs | 10.136 | 1.50×10^-3^ | MDD < HCs | 12.897 | 3.46×10^-4^ | MDD < HCs | 7.821 | 0.005 |
| 60 | 8 | MDD > HCs | 9.849 | 1.75×10^-3^ | MDD < HCs | 11.915 | 5.82×10^-4^ | MDD < HCs | 7.676 | 0.006 |
| 80 | 4 | MDD > HCs | 10.541 | 1.21×10^-3^ | MDD < HCs | 15.999 | 6.85×10^-5^ | MDD < HCs | 9.366 | 0.002 |
| 80 | 6 | MDD > HCs | 10.637 | 1.15×10^-3^ | MDD < HCs | 17.916 | 2.54×10^-5^ | MDD < HCs | 9.727 | 0.002 |
| 80 | 8 | MDD > HCs | 10.624 | 1.16×10^-3^ | MDD < HCs | 17.895 | 2.57×10^-5^ | MDD < HCs | 9.777 | 0.002 |
| 100 | 4 | MDD > HCs | 10.260 | 1.41×10^-3^ | MDD < HCs | 15.999 | 6.85×10^-5^ | MDD < HCs | 9.084 | 0.003 |
| 100 | 8 | MDD > HCs | 10.168 | 1.48×10^-3^ | MDD < HCs | 14.877 | 1.23×10^-4^ | MDD < HCs | 8.998 | 0.003 |

Abbreviations: MDD = major depressive disorder; HCs = healthy controls.

**Supplemental Table S8.** Results of group comparisons on temporal variability, temporal correlation coefficient and characteristic temporal path length in each subset.

| Subset | Temporal variability | | | Temporal correlation coefficient | | | Characteristic temporal path length | | |
| --- | --- | --- | --- | --- | --- | --- | --- | --- | --- |
|  | Comparisons | *F* | FDR-corrected *p* | Comparisons | *F* | FDR-corrected *p* | Comparisons | *F* | FDR-corrected *p* |
| Split-half subset 1 | MDD > HCs | 5.108 | 0.034 | MDD < HCs | 7.288 | 0.022 | MDD < HCs | 4.505 | 0.034 |
| Split-half subset 2 | MDD > HCs | 4.673 | 0.047 | MDD < HCs | 7.296 | 0.022 | MDD < HCs | 3.896 | 0.049 |
| Education-matched subset | MDD > HCs | 5.875 | 0.019 | MDD < HCs | 9.997 | 0.005 | MDD < HCs | 5.557 | 0.019 |

Abbreviations: MDD = major depressive disorder; HCs = healthy controls.

**Supplemental Fig. S1.** The vector characterizing FC between node *k* and all other nodes, which was used to estimate its regional temporal variability (refer to Supplemental Methods for details). FC = functional connectivity.


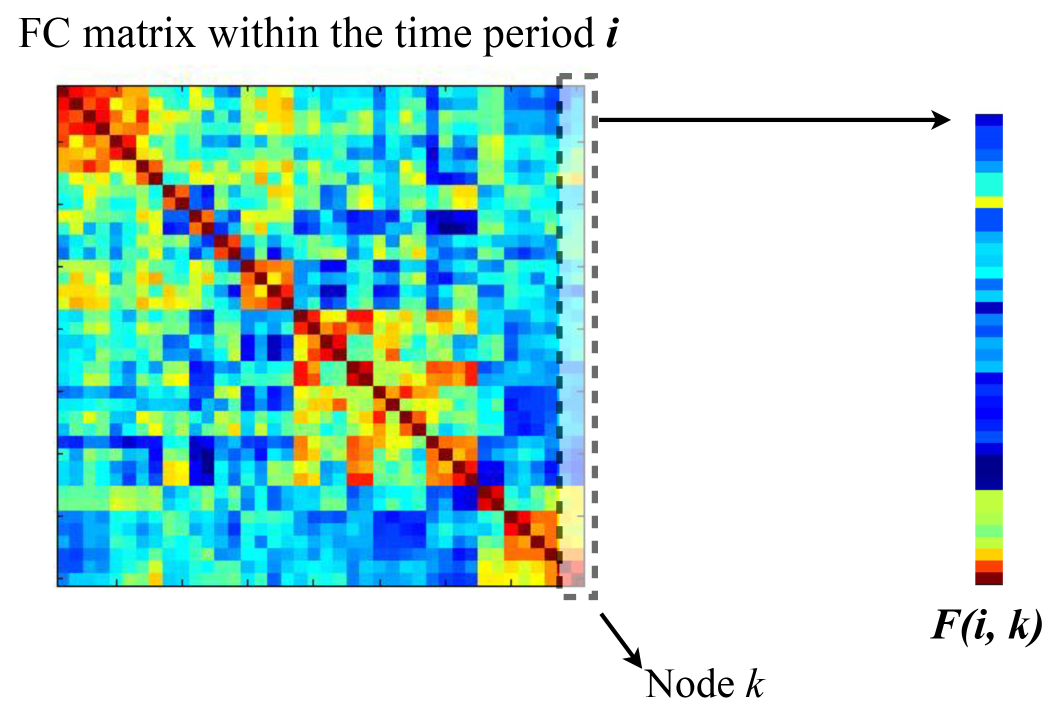


**Supplemental Fig. S2.** In the presented example of a simplified dynamic network, (**A**) the nodal temporal coefficient (*C*) of node *A* is 1, as its neighbors never change over time, and the nodal temporal coefficients of nodes *C* and *D* are both *0* because their neighbors are not overlapped between any two consecutive time points; (**B**) while the nodes *B* and *D* are not directly connected at any time points, they can be contacted in 2 time units via a temporal path (as shown by the red arrows), and therefore their temporal distance (*d*) is 2. Meanwhile, the temporal distance between nodes *B* and *A*, or between nodes *B* and *C* is 1 because they are directly connected.


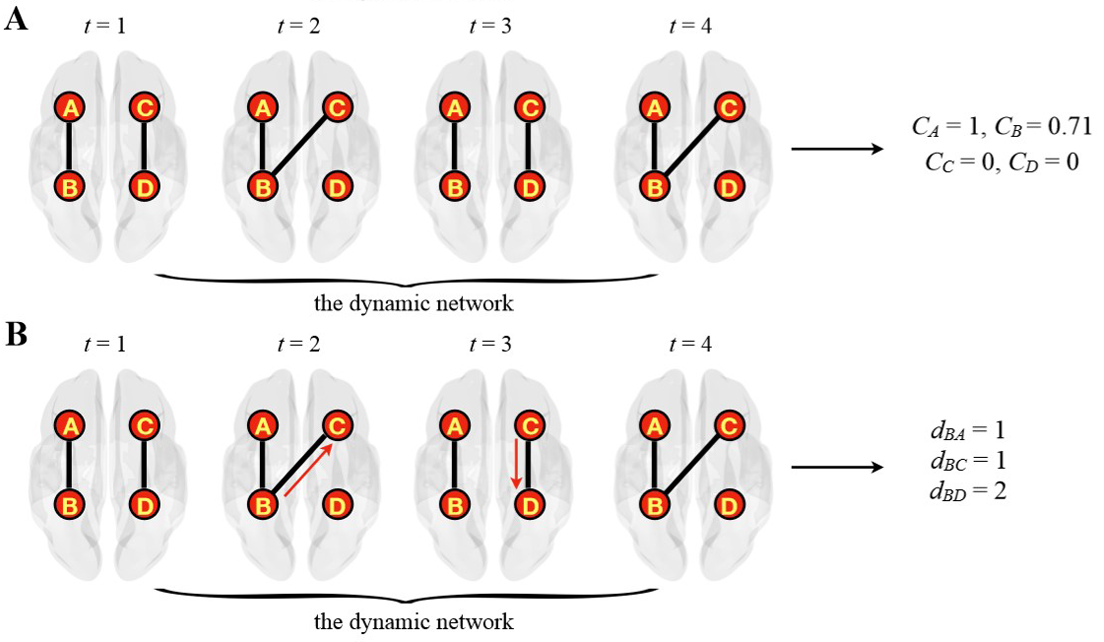


**Supplemental Fig. S3.** Results of partial correlations in each subgroup between each metric and the HAMD score, adjusted for age, gender and site effects. FEDN = first-episode and drug-naïve; HAMD = Hamilton Depression Rating Scale.


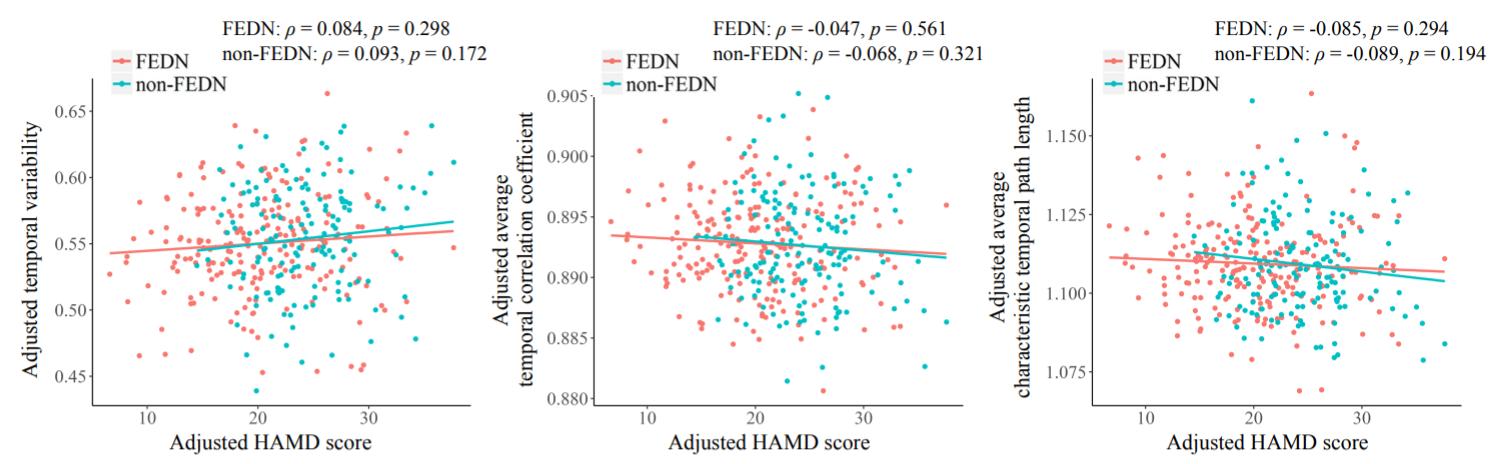


**Supplemental Fig. S4.** Results of group comparisons using the AAL atlas. (**A**) Comparisons at the global level, with FDR-corrected *p* values reported. (**B-D**) Comparisons at the regions level. The nodes showing (**B**) a higher nodal temporal variability, (**C**) a lower nodal temporal correlation coefficient, and (**D**) a shorter nodal temporal path length in MDD patients than HCs were presented (with FDR-corrected *p* < 0.05). ACG = anterior cingulate gyri; ANG = angular gyrus; HC = healthy control; MDD = major depressive disorder; PAL = pallidum; PCUN = precuneus; PUT = putamen; SFGmed = superior frontal gyrus (medial); SPG = superior parietal gyrus; THA = thalamus.


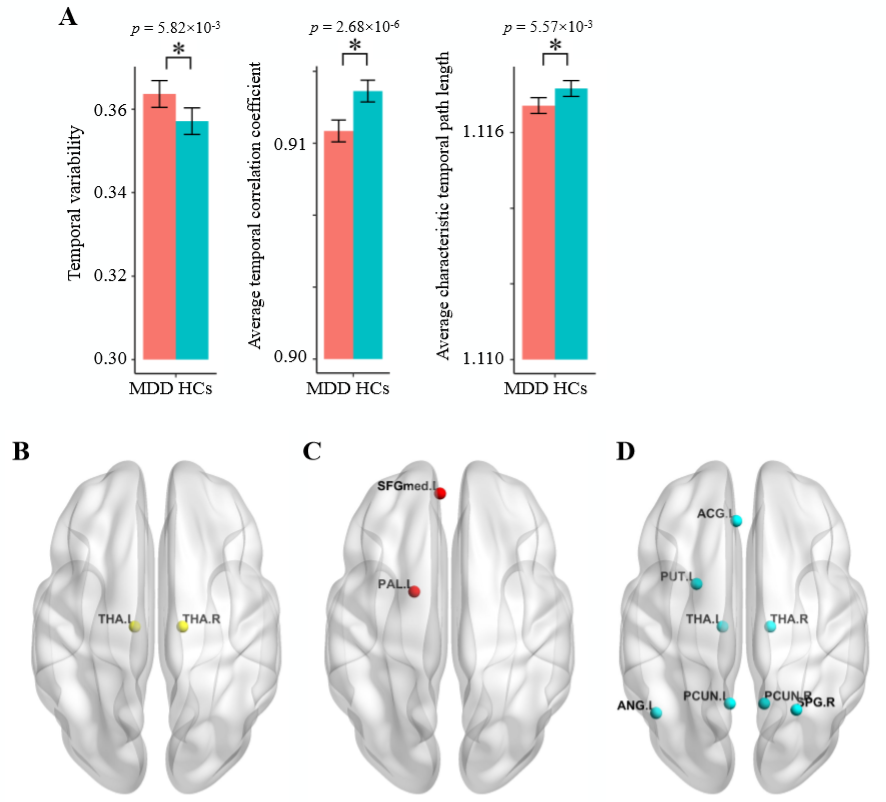


**Supplemental Fig. S5.** Group comparisons on the temporal variability, temporal correlation coefficient and characteristic temporal path length in each individual site. **p* < 0.05. HC = healthy control; MDD = major depressive disorder.


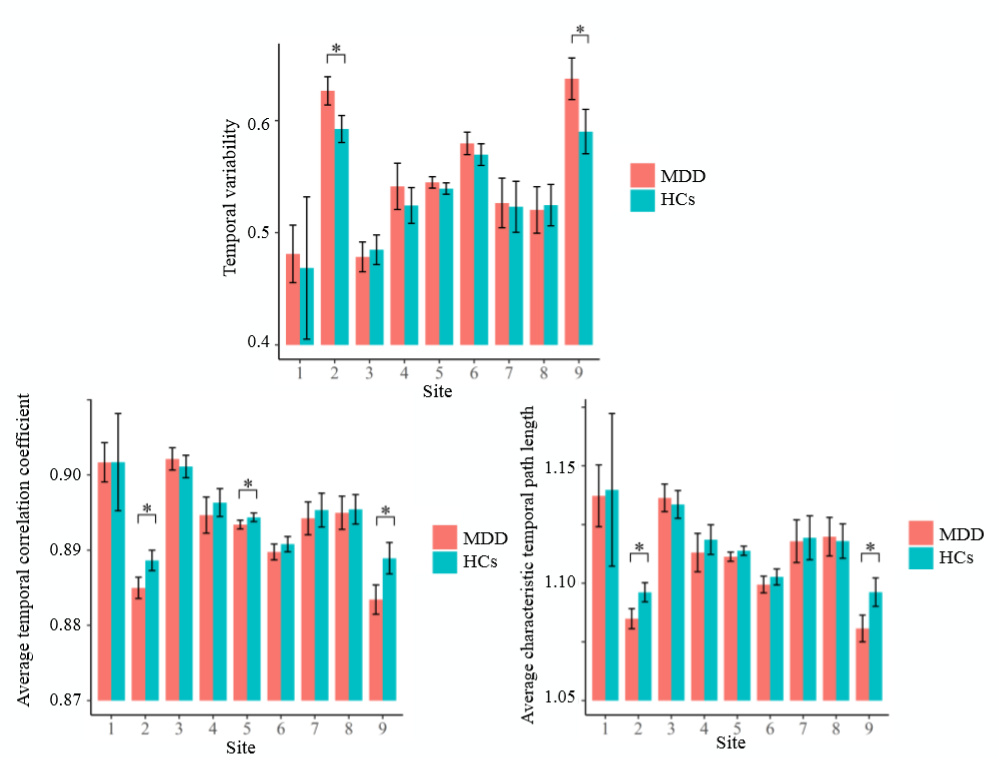

Supplement: Supplementary file 1 [file mmc1.docx]
